# Control of diabetic hyperglycaemia and insulin resistance through TSC22D4

**DOI:** 10.1038/ncomms13267

**Published:** 2016-11-09

**Authors:** Bilgen Ekim Üstünel, Kilian Friedrich, Adriano Maida, Xiaoyue Wang, Anja Krones-Herzig, Oksana Seibert, Anke Sommerfeld, Allan Jones, Tjeerd P. Sijmonsma, Carsten Sticht, Norbert Gretz, Thomas Fleming, Peter P. Nawroth, Wolfgang Stremmel, Adam J. Rose, Mauricio Berriel-Diaz, Matthias Blüher, Stephan Herzig

**Affiliations:** 1 Institute for Diabetes and Cancer (IDC), Helmholtz Center Munich, and Joint Heidelberg-IDC Translational Diabetes Program, Internal Medicine I, 85764 Neuherberg, Germany; 2 Department of Internal Medicine IV, Heidelberg University Hospital, 69120 Heidelberg, Germany; 3 Center for Clinical Research, Medical Faculty Mannheim, 68167 Mannheim, Germany; 4 Department of Medicine I and Clinical Chemistry, Heidelberg University, 69120 Heidelberg, Germany; 5 Department of Medicine, University of Leipzig, 04103 Leipzig, Germany

## Abstract

Obesity-related insulin resistance represents the core component of the metabolic syndrome, promoting glucose intolerance, pancreatic beta cell failure and type 2 diabetes. Efficient and safe insulin sensitization and glucose control remain critical therapeutic aims to prevent diabetic late complications Here, we identify transforming growth factor beta-like stimulated clone (TSC) 22 D4 as a molecular determinant of insulin signalling and glucose handling. Hepatic TSC22D4 inhibition both prevents and reverses hyperglycaemia, glucose intolerance and insulin resistance in diabetes mouse models. TSC22D4 exerts its effects on systemic glucose homeostasis—at least in part—through the direct transcriptional regulation of the small secretory protein lipocalin 13 (LCN13). Human diabetic patients display elevated hepatic *TSC22D4* expression, which correlates with decreased insulin sensitivity, hyperglycaemia and LCN13 serum levels. Our results establish TSC22D4 as a checkpoint in systemic glucose metabolism in both mice and humans, and propose TSC22D4 inhibition as an insulin sensitizing option in diabetes therapy.

## Abstract (web summary)

TSC22D4 regulates hepatic lipoprotein production, but has so far mainly been studied in the context of cancer cachexia. Here, the authors show TSC22D4 inhibition improves insulin sensitivity in several mouse models of diabetes, which they attribute at least in part to the induction of secreted LCN13.

## Graphical abstract

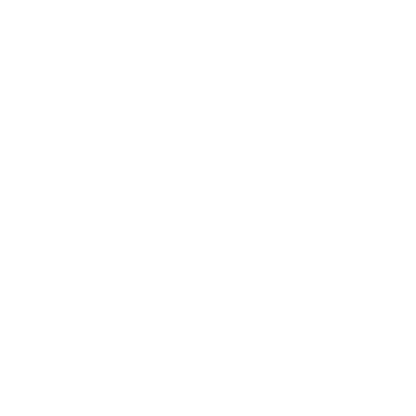

Under physiological conditions, energy homeostasis is tightly controlled by counter-regulatory hormonal systems. Whereas pancreatic glucagon as well as adrenal glucocorticoid hormones mediate essential parts of the fasting response to ensure organ function during nutrient depletion^1^, the pancreatic beta-cell hormone insulin triggers the fast uptake and oxidative catabolism of glucose in liver, muscle and adipose tissue, and simultaneously inhibits glycogenolysis and gluconeogenesis in liver during the postprandial state^2^. Insulin actions are mediated through its membrane-bound tyrosine kinase receptor. Upon insulin binding, the intrinsic tyrosine kinase activity of the insulin receptor at the cell surface becomes activated and leads to the subsequent tyrosine phosphorylation of multiple signalling components, involving consecutive phosphorylation events at the insulin receptor substrate proteins and the subsequent activation of the phosphatidylinositol 3 kinase (PI3K)/protein kinase B (PKB/Akt) signalling axis^3^,^4^. Upon insulin stimulation 3-phosphoinositide-dependent protein kinase-1 (PDK1) phosphorylates PKB/Akt on T308 within the kinase domain, whereas PDK2, which is represented by several kinases including mechanistic target of rapamycin complex 2 (mTORC2) phosphorylates Akt within its C terminus tail on S473 (refs 5, 6). Once phosphorylated and activated Akt phosphorylates and inhibits the inhibitor of mTORC1 the tuberous sclerosis complex 2 (Tsc2; a.k.a Tuberin) within the Tsc1/2 to activate downstream mTORC1 signalling to S6K1 and S6. Other targets of Akt include glycogen synthase kinase (GSK) 3 beta and *Forkhead* Box O 1 and 3 (FoxO1 and 3) transcription factors^7^. Insulin signal transduction exerts control over biochemical pathways through either modulation of metabolic key enzyme activities or through the stimulation or inhibition of metabolic target gene transcription, eventually culminating in the regulation of anabolic glucose, lipid and protein metabolism^8^.

The inability of key metabolic tissues, including skeletal muscle, adipose tissue and liver to respond to normal circulating concentrations of insulin, that is, insulin resistance, is commonly associated with obesity, ageing and a sedentary lifestyle^8^. Under these conditions, systemic glucose and lipid homeostasis are substantially compromised, leading to successive pancreatic beta cell exhaustion and further deterioration of glucose homeostasis. Upon the manifestation of glucose intolerance as a marker of the pre-diabetic state, 5–10% of glucose-intolerant patients indeed further progress into full-blown type 2 diabetes^9^.

Interestingly, insulin resistance is not only associated with obesity-related metabolic dysfunction but also with opposing conditions of systemic energy availability, for example, tumour-induced body wasting (cancer cachexia) and lipodystrophy. Indeed, both excess and lack of adipose tissue energy stores are coupled to systemic inflammation, fatty liver development, skeletal muscle degradation and increased adipose tissue lipolysis, favoured by insulin resistance and the lack of anabolic insulin action^10^. Consequently, safe and effective modalities to improve systemic insulin sensitivity are direly needed not only for counteracting obesity-related type 2 diabetes but may also pave the way for novel therapeutic approaches in wasting diseases.

In this respect, we have recently identified transcription factor TSC22D4 as a regulator of hepatic lipid metabolism during cancer cachexia^12^. Livers of tumour-bearing mice had elevated levels of TSC22D4 and hepatic knockdown of TSC22D4 acutely led to the induction of lipogenic genes. Interestingly, hepatic TSC22D4 levels positively correlated with the degree of tumour-induced body wasting^12^, overall suggesting that hepatic TSC22D4 activity may not only be involved in the control of tissue-specific lipid metabolism but also be linked to systemic energy homeostasis by thus far unknown mechanisms. This hypothesis prompted us to define the hepatic TSC22D4 target gene network and the systemic implications of hepatic TSC22D4 action at a more global level. We now identify the hepatic TSC22D4 as a potent regulator of insulin sensitivity in both murine and human diabetes, acting—at least in part—through the secreted factor lipocalin LCN13. TSC22D4 thus represents a previously unknown checkpoint in inter-organ communication and systemic metabolic control and may serve as an attractive target in insulin sensitizing diabetes therapies.

## Results

### Hepatic TSC22D4 deficiency improves insulin sensitivity

To understand the range of TSC22D4-regulated transcriptional programmes beyond the lipogenic pathway, we performed high throughput transcriptome profiling in primary mouse hepatocytes infected with adenovirus carrying either non-specific control or TSC22D4-specific shRNA. KEGG pathway analysis demonstrated that loss of TSC22D4 significantly regulated several biological processes, most notably including insulin signalling (Supplementary Fig. 1a), suggesting that TSC22D4 may exert an important impact on hepatic and systemic insulin responses.

**Figure 1:**
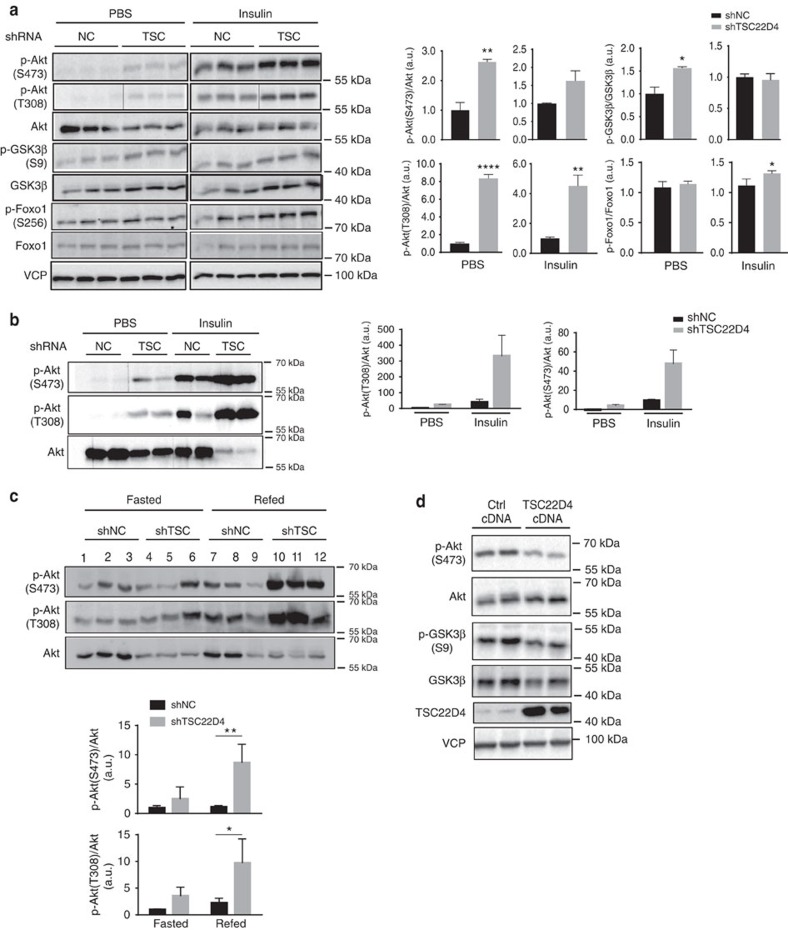
TSC22D4 controls intra-hepatic insulin signalling. (**a**) Western blot analysis from representative negative control (NC shRNA) or TSC22D4 (TSC shRNA) shRNA adenovirus-treated primary mouse hepatocytes using indicated antibodies. Right: quantification of the phospho-immunoblots normalized to corresponding total protein levels (*y* axis is in arbitrary units). Statistical analysis: Student's *t*-test. *P≤0.05; **P≤0.01; ***P≤0.001. Error bars indicate standard error of the mean (s.e.m.). (**b**) Western blot analysis of liver extracts from representative control (shNC) or TSC22D4 (shTSC) shRNA adenovirus-injected C57Bl/6 mice 7 days after injection using indicated antibodies. Right: quantification of the phospho-immunoblots normalized to corresponding total protein levels (*y* axis is in arbitrary units). Animals were injected i.p. with either PBS or insulin 20 min before sacrifice. (**c**) Western blot analysis of liver extracts from 16 h fasted or 30 min refed control (shNC) or TSC22D4 (shTSC) shRNA adenovirus-injected C57Bl/6 mice 7 days after injection using indicated antibodies. Bottom: Quantification of the phospho-immunoblots normalized to corresponding total protein levels (*y* axis is in arbitrary units). Statistical analysis: two way ANOVA with multiple comparisons test; **P≤0.01; ****P≤0.0001. Error bars in **b**,**c**, indicate standard deviation (s.d). (**d**) Western blot analysis of liver extracts from representative empty control (ctrl cDNA) or Flag-TSC22D4 cDNA adenovirus-injected C57Bl/6 mice 7 days after injection using indicated antibodies. Following 18 h of fasting, mice were refed for 6 h before sacrifice.

To test this hypothesis, we employed TSC22D4-deficient and control primary mouse hepatocytes and probed for the phosphorylation of prototypical insulin signalling pathway components. Indeed, TSC22D4 depletion significantly increased phosphorylation of Akt/PKB on both T308 and S473 as well as its downstream targets GSK3 beta on S9 and FoxO1 on S256 under basal and insulin-stimulated conditions, respectively (Fig. 1a), overall indicating that TSC22D4 downregulates the insulin signalling pathway in a hepatocyte-autonomous manner.

Furthermore, liver-specific knockdown of TSC22D4 in wild-type animals (Supplementary Fig. 1b,c) enhanced the hepatic Akt T308/S473 phosphorylation in response to either exogenous insulin injection (Fig. 1b) or re-feeding in a fasting–feeding regimen (Fig. 1c), while adenoviral liver-specific overexpression of TSC22D4 robustly impaired S473 phosphorylation of Akt in mouse liver and also impaired the Akt signalling to its downstream targets, including GSK3 beta (Fig. 1d; Supplementary Fig. 1f), demonstrating that hepatic TSC22D4 is a critical determinant of insulin signalling also *in vivo*.

**Figure 2:**
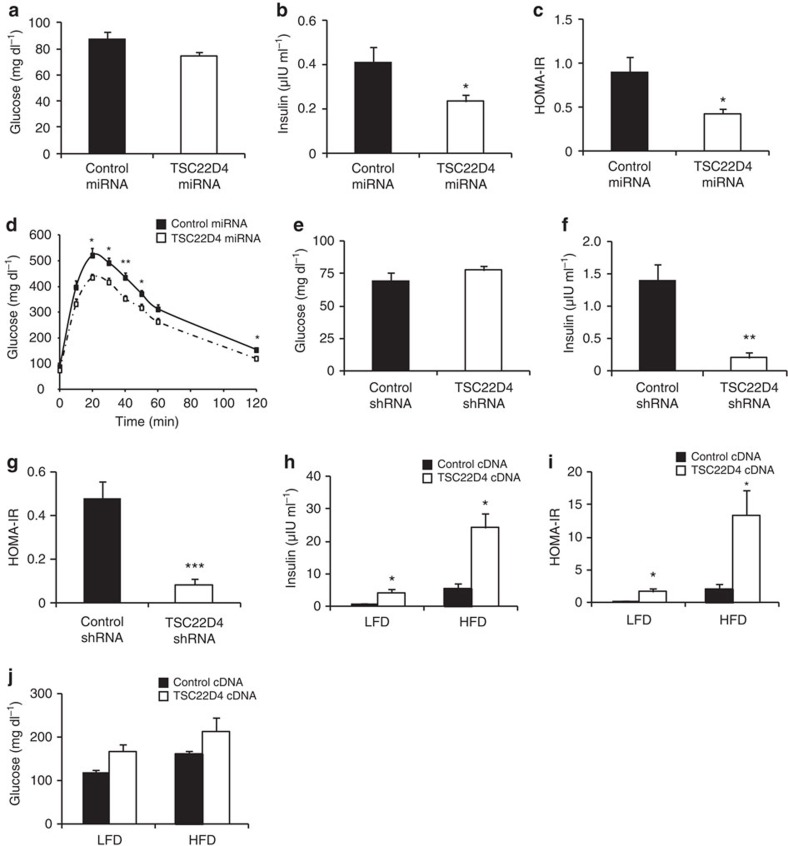
TSC22D4 regulates systemic glucose homeostasis. (**a**) Serum glucose levels of 16 h fasted control or TSC22D4 miRNA AAV–injected C57Bl/6 mice 6 weeks after virus injection (means±s.e.m., *n*=5). (**b**) Serum insulin levels in same mice as in **a**. (**c**) HOMA-IR index of same mice as in **a**. (**d**) Glucose tolerance test in same mice as in **a**, 2 weeks after miRNA injection. Glucose was injected i.p. at a concentration of 1 g glucose kg^−1^ body weight (means±s.e.m., *n*≥6). (**e**) Serum glucose levels of 16 h fasted control or TSC22D4shRNA adenovirus–injected C57Bl/6 mice 7 days after virus injection (means±s.e.m., *n*≥6). (**f**) Serum insulin levels in same mice as in **e**. (**g**) HOMA-IR index in same mice as in **e**. (**h**) Serum insulin levels in empty control (control cDNA) or Flag- TSC22D4 (TSC22D4 cDNA) cDNA adenovirus-injected random fed C57Bl/6 mice that were fed either a LFD or HFD for 11 weeks (means±s.e.m., *n*=5). (**i**) HOMA-IR index in same mice as in **h**. (**j**) Serum glucose levels in same mice as in **h**. Statistical analysis: Student's *t*-test, *P≤0.05; **P≤0.01; ***P≤0.001.

These results prompted us to explore the functional impact of hepatic TSC22D4 on systemic glucose metabolism in healthy C57Bl/6 mice. Thus, we employed an adeno-associated virus (AAV) delivery system allowing the expression of TSC22D4-directed miRNAs specifically in liver parenchymal cells but not in other liver cell types for a period of several months^13^. Six weeks after AAV-mediated miRNA delivery, wild-type mice with hepatocyte-specific TSC22D4 deficiency (Supplementary Fig. 2a) showed no change in fasting blood glucose levels (Fig. 2a) but displayed significantly reduced levels of serum insulin (Fig. 2b) and an improved homeostasis model of assessment-insulin resistance (HOMA-IR) index as compared with control littermates (Fig. 2c). Indeed, hepatic TSC22D4 deficiency improved insulin-triggered systemic glucose clearance in an intra-peritoneal insulin tolerance test (Supplementary Fig. 2b), and also improved glucose tolerance (Fig. 2d), overall pointing towards improved systemic insulin sensitivity and glucose handling upon hepatocyte-specific TSC22D4 knockdown. To validate these findings in an independent and more acute setting, we down regulated hepatic TSC22D4 expression by adenoviral shRNA delivery into wild-type mice. In agreement with the long-term effects, inhibition of hepatic TSC22D4 had no effect on serum glucose concentrations in healthy animals under both fasting and feeding conditions (Fig. 2e; Supplementary Fig. 2c), but again reduced fasting and feeding serum insulin (Fig. 2f; Supplementary Fig. 2d) and HOMA-IR in comparison to control shRNA-injected littermates (Fig. 2g). In line with the negative regulatory impact of TSC22D4 on hepatic insulin signalling (Fig. 1d), liver-specific overexpression of TSC22D4 significantly elevated serum insulin levels in both low-fat diet (LFD) (10% calories from fat) and high-fat diet (HFD) (60% calories from fat)-fed C57Bl/6 mice as compared with control littermates (Fig. 2h), correlating with increased HOMA-IR under these conditions (Fig. 2i), and a trend to elevate serum glucose levels (Fig. 2j).

### Hepatic TSC22D4 knockdown prevents diabetic hyperglycaemia

**Figure 3:**
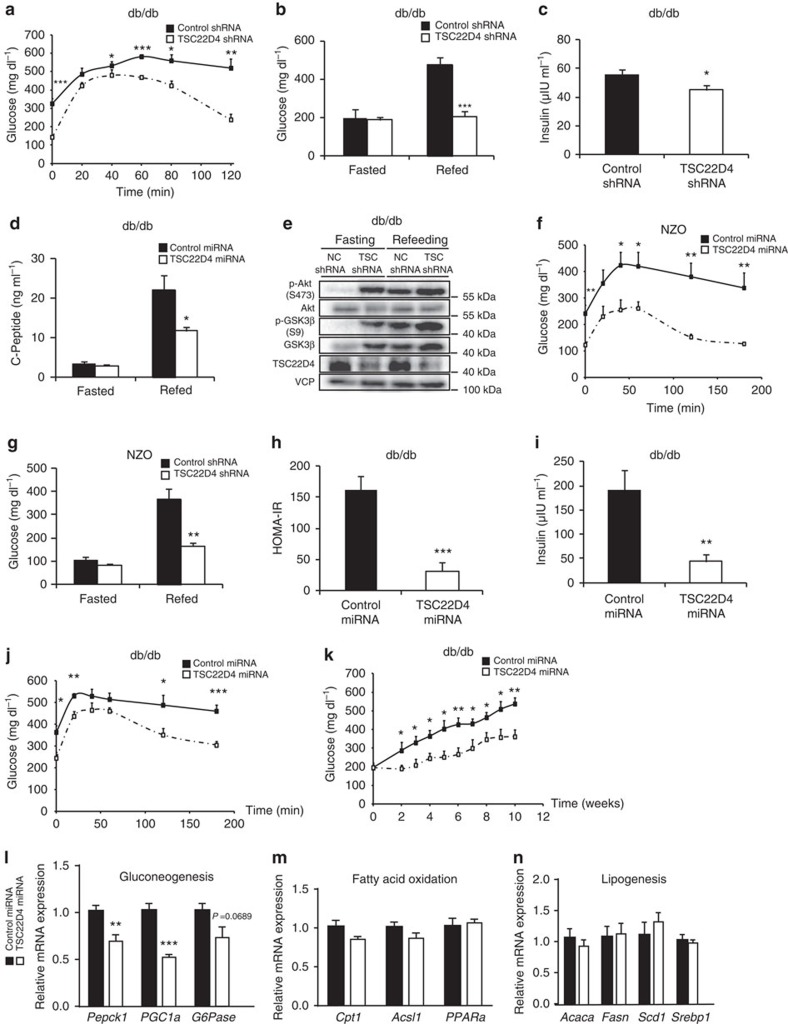
TSC22D4 deficiency counteracts diabetic hyperglycaemia and insulin resistance. (**a**) Glucose tolerance test in control or TSC22D4 shRNA adenovirus–injected db/db mice 1 week after injection. Glucose was injected i.p. at a concentration of 1 g glucose kg^−1^ body weight (means±s.e.m., *n*≥6). (**b**) Serum glucose levels in same mice as in **a** after 16 h fasting and refeeding. (**c**) Serum insulin levels in same mice as in **a**. (**d**) Serum C-Peptide levels in same mice as in **a** after 16 h fasting and refeeding. (**e**) Representative western blot of liver extracts from control (NC shRNA) or TSC22D4 (TSC shRNA) shRNA adenovirus–injected db/db mice; same mice as in **a** using indicated antibodies. Blot representative of 6 similar samples. (**f**) Glucose tolerance test in control or TSC22D4 shRNA adenovirus–injected NZO mice 1 week after injection. Glucose was i.p. injected at a concentration of 1 g glucose kg^−1^ body weight (means±s.e.m., *n*≥6). (**g**) Serum glucose levels in same mice as in **f** after 16 h fasting and refeeding. (**h**) HOMA-IR index in control or TSC22D4 miRNA AAV–injected (at 5 weeks of age) db/db mice 4 weeks after miRNA injection (means±s.e.m., *n*≥6). (**i**) Serum insulin levels in same mice as in **h** 10 weeks after miRNA injection. (**j**) Glucose tolerance test in the same mice as in **h** 4 weeks after miRNA injection. Glucose was injected i.p at a concentration of 1 g glucose kg^−1^ body weight. (**k**) Weekly serum glucose quantification of same mice as in **h**. (**l**–**n**) Quantitative PCR analysis of phosphoenolpyruvate carboxykinase (*Pepck1*), peroxisome proliferator-activated receptor gamma coactivator 1-alpha (*Pgc-1alpha*), glucose-6 phosphatase (*G6Pase*), carnitine palmitoyltransferase I (*Cpt1*), acyl-CoA synthetase long-chain family member 1 (*Acsl1*), peroxisome proliferator-activated receptor alpha (*PPARalpha*), acetyl-CoA carboxylase alpha (*Acaca*), fatty acid synthase (*Fasn*), stearoyl-coA desaturase-1 (*Scd1*), sterol regulatory element-binding protein 1 (*Srebp1*) in livers of same mice as in **h** 10 weeks after miRNA injection. Error bars in **l**−**n** represent standard deviation (s.d.). Statistical analysis: Student's *t*-test, *P≤0.05; **P≤0.01; ***P≤0.001.

Our results thus far demonstrated that hepatic TSC22D4 inhibition (a) improves systemic insulin sensitivity and glucose tolerance without causing episodes of hypoglycaemia under healthy conditions (Fig. 2a) and (b) that TSC22D4 also regulates insulin sensitivity under conditions of (high-fat diet induced) metabolic dysfunction (Fig. 2i). Thus, we next tested the hypothesis that hepatic TSC22D4 inhibition could serve as a novel approach to counteract diabetic hyperglycaemia and glucose intolerance. In this respect, we acutely downregulated hepatic TSC22D4 expression in 14 weeks old db/db mice, a standard model for insulin-resistant obesity and type 2 diabetes^14^. Remarkably, already 7 days after adenovirus delivery, liver TSC22D4 deficiency improved insulin (Supplementary Fig. 3a) and glucose tolerance as compared with control littermates (Fig. 3a), and also normalized serum blood glucose levels in diabetic animals under refed conditions (Fig. 3b). In line with the anti-diabetic action of TSC22D4 downregulation, water intake (Supplementary Fig. 3b) as well as serum levels of insulin (Fig. 3c) and c-peptide (Fig. 3d) were substantially diminished in TSC22D4-deficient mice as compared with controls, correlating with an induction of S473 and S9 phosphorylation of hepatic Akt and GSK3 beta, respectively, as well as increased liver glycogen levels (Fig. 3e; Supplementary Fig. 3c). Of note, short-term hepatic TSC22D4 inhibition had no effect on body weight (Supplementary Fig. 3d) and lowered serum alanine aminotransferase levels (Supplementary Fig. 3e) indicating the absence of liver toxicity under these conditions.

To confirm the gluco-regulatory function of hepatic TSC22D4 in an independent model, we acutely disrupted TSC22D4 expression in livers of New Zealand obese (NZO) mice, which represent a multigenic model of diabesity^15^. At 16 weeks of age, NZO mice displayed severe obesity, insulin resistance and hyperglycaemia as described^15^. Liver-specific knockdown of TSC22D4 markedly improved insulin (Supplementary Fig. 3f) and glucose tolerance (Fig. 3f), normalized blood glucose levels under refed conditions (Fig. 3g), and elevated Akt and GSK3 beta phosphorylation (Supplementary Fig. 3j) compared with control littermates, perfectly matching the effects in db/db mice (Fig. 3a–e). In addition, while TSC22D4 knockdown in NZO mice did not affect body weight, it slightly decreased abdominal and inguinal WAT mass (Supplementary Fig. 3g–i).

The efficient and acute improvements in diabetic glucose homeostasis next prompted us to test for a putative diabetes prevention potential of hepatic TSC22D4 inhibition. Therefore, we chronically knocked-down TSC22D4 specifically in hepatocytes of young (5 weeks old) db/db mice that still showed no signs of obesity and metabolic dysfunction. Remarkably, while control animals developed severe hyperglycaemia over the course of 10 weeks and displayed substantial insulin resistance, hyperinsulinemia and glucose intolerance at an age of 15 weeks (Fig. 3h-j), hepatic TSC22D4 knockdown (Supplementary Fig. 3k) not only prevented the onset of hyperglycaemia (Fig. 3k) but also significantly improved insulin sensitivity and insulin tolerance (Fig. 3h,i; Supplementary Fig. 3k–m) and glucose tolerance over time (Fig. 3j). Noteworthy, at the end of the experimental 10-week period, TSC22D4 knockdown had caused a roughly 25% reduction in HbA1c levels as compared with controls (Supplementary Fig. 3n), thereby outpacing the performance of metformin in an almost identical db/db experimental setup^16^. In contrast to the induction of hypertriglyceridemia in cancer cachectic wild-type mice upon liver-specific TSC22D4 knockdown^12^, long-term TSC22D4 deficiency diminished serum triglycerides (TAGs) (Supplementary Fig. 3o) but left liver TAG levels unaltered (Supplementary Fig. 3p). Only minor changes in body weight, fat, and lean mass were transiently detectable over the course of the experiment (Supplementary Fig. 3r–t). Consistent with these metabolic phenotypes, long-term TSC22D4 knockdown impaired gluconeogenic gene expression but left genes involved in fatty acid oxidation and lipogenesis mostly unaffected (Fig. 3l–n).

### LCN13 partially mediates the effects of hepatic TSC22D4

**Figure 4:**
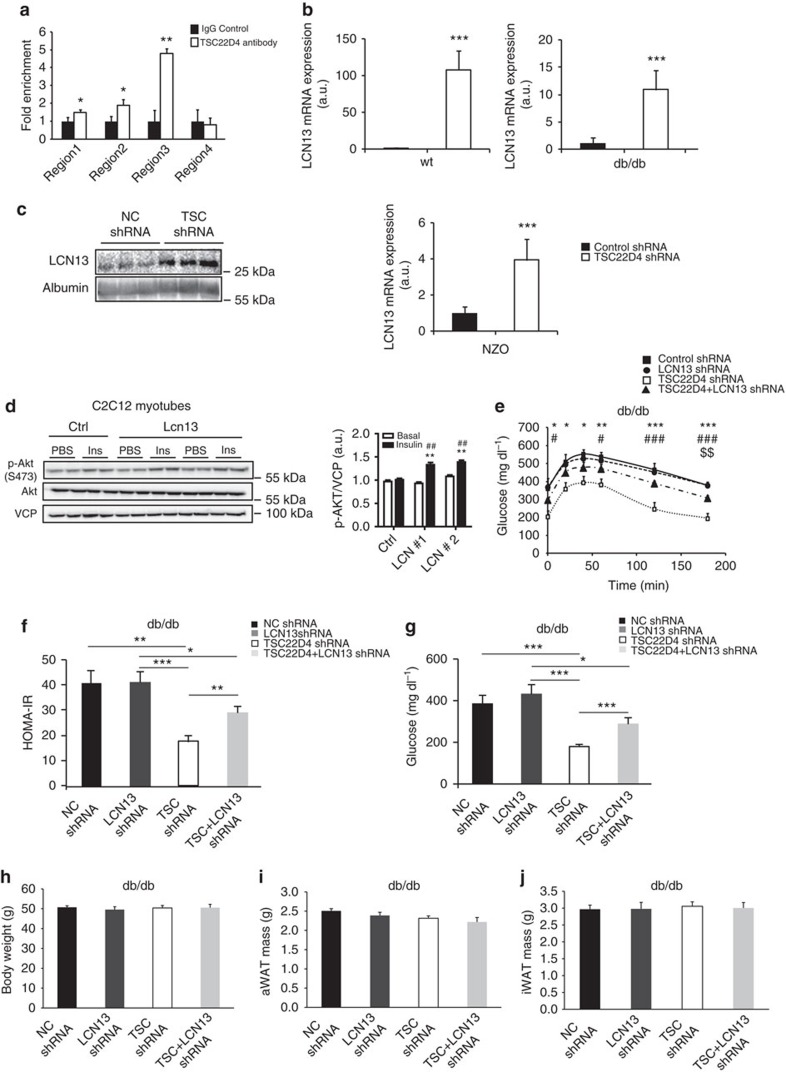
TSC22D4 acts via the LCN13 endocrine system. (**a**) Chromatin immunoprecipitation of LCN13 promoter regions (1–3) by antibodies against TSC22D4 in livers of wild-type mice. Fold enrichment relative to negative isotype control IgG is determined by qPCR. Region 4 represents a negative PCR control (*n*=2–3). Similar results were obtained by using a different TSC22D4 antibody. (**b**) Quantitative PCR analysis of LCN13 in livers of control or TSC22D4 shRNA adenovirus-injected wild-type C57Bl/6 (left), db/db (middle), and NZO mice (below) (means± s.e.m., *n*≥ 6 for each experiment). (**c**) Serum from control (NC shRNA) or TSC22D4 (TSC shRNA) shRNA adenovirus–injected C57Bl/6 mice 7 days after injection was immunoblotted with LCN13 antibody. Albumin antibody was used as loading control. (**d**) Representative western blot from control (PBS) or LCN13 (200 nM)-treated C2C12 myotube extracts using indicated antibodies. LCN13 (3 h) and insulin treatment for 15 min (30 nM) indicated, respectively. Right: densitometric analysis shown. **Indicates effect of insulin; ^##^Indicates effect of LCN13. (**e**) Glucose tolerance test in control (control shRNA), LCN13 (LCN13 shRNA), TSC22D4 (TSC22D4 shRNA), TSC22D4 plus LCN13 (TSC22D4 +LCN13 shRNA) shRNA adenovirus–injected db/db mice 1 week after injection. Glucose was injected i.p. at a concentration of 1 g glucose kg^−1^ body weight. *Indicates significance between NC and TSC22D4 group; ^#^Indicates significance between TSC22D4 and TSC22D4 +LCN13 group; ^$^Indicates significance between NC and TSC22D4 +LCN13 group, (means±s.e.m., *n*≥6). (**f**) HOMA-IR index in same mice as in **e**. (**g**) Serum glucose levels in same mice as in **e**. (**h**) Body weight of mice as in **e**. (**i**) Abdominal white adipose tissue (aWAT) mass in same mice as in **e**. (**j**) Inguinal white adipose tissue (iWAT) in same mice as in **e**. Statistical analysis: Student's *t*-test, *P≤0.05; **P≤0.01; ***P≤0.001, ^#^*P*≤0.05; ^##^*P*≤0.01; ^###^*P*≤0.001, ^$^*P*≤0.05; ^$$^*P*≤0.01; ^$$$^*P*≤0.001.

To get mechanistic insights into the insulin sensitizing and glucose lowering action of hepatic TSC22D4, we screened for the most strongly regulated TSC22D4 target genes in the transcriptome data sets from TSC22D4-deficient and control mice (Supplementary Fig. 4a). Remarkably, the most strongly upregulated gene upon TSC22D4 knockdown was represented by lipocalin 13 (LCN13), a member of a conserved family of small secreted proteins^17^. Liver cistrome analysis by chromatin immunoprecipitation experiments coupled to massive parallel sequencing (ChIP-seq) and subsequent ChIP-PCR confirmation identified three TSC22D4-binding sites within the *Lcn 13* gene locus (Fig. 4a), proposing a direct regulatory function of TSC22D4 in *Lcn 13* expression through chromatin recruitment. Indeed, siRNA-mediated TSC22D4 knockdown in hepatoma cells transfected with *Lcn 13* luciferase reporter constructs carrying the identified TSC22D4-binding sites within the *Lcn 13* locus induced reporter gene activities (Supplementary Fig. 4b). In accordance, hepatic TSC22D4 knockdown elevated *Lcn 13* mRNA levels 100-, 11- and 4-fold in livers of wild-type, db/db, as well as NZO mice, respectively (Fig. 4b). Also, *Lcn 13* mRNA levels were significantly diminished upon hepatic TSC22D4 overexpression under both LFD and HFD conditions (Supplementary Fig. 4c), and *Lcn 13* mRNA was also suppressed in livers of methionine- and cholin-deficient (MCD) diet-fed lipodystrophic mice (Supplementary Fig. 4d), thereby correlating with high levels of TSC22D4 in this model as reported previously^12^. In line with the proposed endocrine function of *Lcn 13*, western blot analysis of Akt S473 phosphorylation in skeletal muscle revealed that hepatic TSC22D4 deficiency induced not only hepatic but also skeletal muscle insulin signalling (Supplementary Fig. 4e), thereby correlating with elevated LCN13 serum levels upon hepatic TSC22D4 deficiency (Fig. 4c) and with direct insulin sensitization in cultured C2C12 myotubes as well as differentiated 3T3-L1 adipocytes upon exposure to recombinant LCN13 (Fig. 4d, data not shown). Indeed, hepatic TSC22D4 knockdown did not only elevate liver but also skeletal muscle glycogen stores (Supplementary Fig. 4f). To address the functional role of LCN13 as a potential mediator of hepatic TSC22D4-dependent glucose control, we performed genetic rescue experiments in db/db diabetic mice. Single TSC22D4 knockdown significantly ameliorated glucose intolerance, insulin resistance and hyperglycaemia in db/db mice as shown above (Fig. 4e–g). Remarkably simultaneous knockdown of hepatic LCN13 and TSC22D4 expression by double adenoviral shRNA delivery (Supplementary Fig. 4g) partially re-induced systemic glucose intolerance (Fig. 4e) as well as dysfunctional glucose homeostasis and insulin resistance in diabetic mice (Fig. 4f,g). Consistently, simultaneous knockdown of LCN13 and TSC22D4 also abrogated the improved liver and skeletal muscle insulin signalling that we observed with single TSC22D4 knockdown, indicated by Akt S473 phosphorylation and Glut4 expression (Supplementary Fig. 4h,i), while levels of pro-inflammatory cytokines were unaltered (Supplementary Fig. 4j,k). Taken together, these findings demonstrate that inhibition of LCN13 acts as one major functional downstream target of TSC22D4 in the control of (diabetic) glucose intolerance and insulin resistance *in vivo*. In addition neither TSC22D4 nor LCN13 single or double knockdowns affected body weight, abdominal or inguinal WAT mass compared with control animals (Fig. 4h–j). Indeed, consistent with improvements in insulin sensitivity and glucose tolerance by whole-body transgenic overexpression of LCN13 (ref. 18) or hepatic TSC22D4 knockdown, comparative transcriptome profiling in control, single LCN13- and double TSC22D4/LCN13-deficient animals revealed that TSC22D4-dependent hepatic LCN13 regulation significantly controlled gluco- and lipid-regulatory processes as defined by KEGG pathway and gene clustering analyses in skeletal muscle (Supplementary Fig. 5a,b), overall suggesting that the TSC22D4-dependent effects on systemic insulin sensitivity and glucose handling were mediated through both local, hepatic and remote, LCN13-dependent effects on extra-hepatic tissues, including skeletal muscle and probably adipose tissue. Of note, LCN13 re-constitution in TSC22D4 knockdown animals (Fig. 4) only partially reversed the TSC22D4-mediated phenotype and previous reports demonstrated that TSC22D4 localizes both in the nucleus and in the cytoplasm in cultured neuronal cells^19^, suggesting that in addition to the nuclear regulation of *LCN13* gene expression, TSC22D4 may engage additional cytoplasmic mechanisms to control cellular insulin signalling. This hypothesis is in line with the cell-autonomous effects of TSC22D4 inhibition on insulin signalling in isolated hepatocytes (Fig. 1) and needs to be further explored in future studies.

### *Tsc22d4* levels correlate with insulin sensitivity in humans

**Figure 5:**
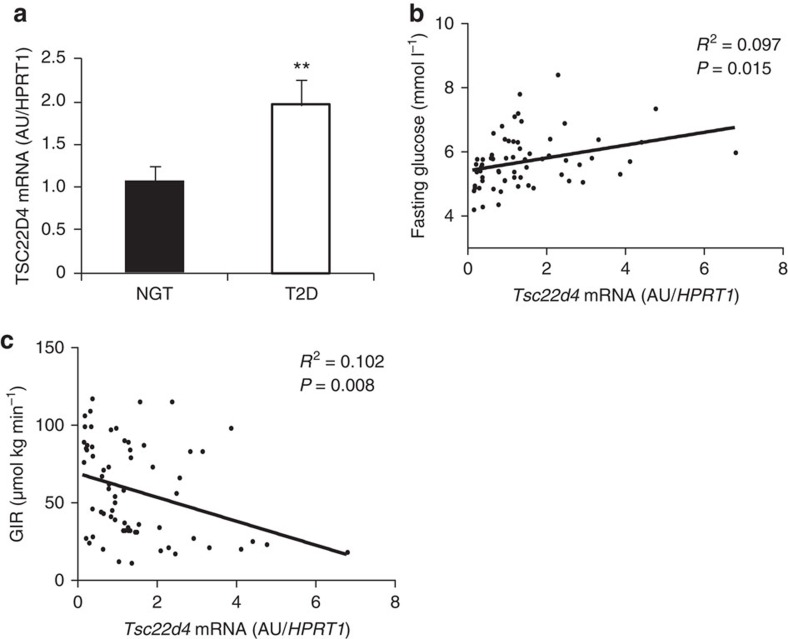
TSC22D4 levels correlate with insulin sensitivity in humans. (**a**) Quantitative PCR analysis of *Tsc22d4* mRNA expression in livers of patients with type 2 diabetes (T2D, *n*=26) or normal glucose tolerance (NGT, *n*=40) Error bars indicate standard error of the mean (s.e.m.). (**b**) Correlation of hepatic expression of *Tsc22d4* mRNA and fasting plasma glucose in the same patients as in **a**. (**c**) Correlation of human liver expression of *Tsc22d4* mRNA and glucose infusion rate (GIR) during hyperinsulemic-euglycaemic clamp study in the same patients as in **a**. Statistical analysis for **a**: Student's *t*-test, **b** and **c**: Pearson correlation coefficient, *P≤0.05; **P≤0.01; ***P≤0.001.

To finally test the relevance of our findings in humans, we analysed a cohort of 66 patients with normal glucose tolerance (NGT) or type 2 diabetes (T2D) (Supplementary Table 1). As shown in Fig. 5a, *TSC22D4* mRNA was significantly elevated in livers of T2D patients as compared with NGT counterparts. Consistent with the insulin-sensitizing and gluco-regulatory functions of TSC22D4 in mice, hepatic *Tsc22d4* mRNA levels significantly correlated with fasting glucose levels in humans (Fig. 5b). Furthermore, *Tsc22d4* expression negatively correlated with insulin sensitivity across this human cohort, the latter determined by the glucose infusion rate (GIR) during a hyperinsulinemic-euglycaemic clamp (Fig. 5c). *Tsc22d4* mRNA levels positively correlated with circulating TAG and pro-inflammatory cytokine levels (Supplementary Fig. 5c,d) thereby further supporting our results from animal models (Supplementary Fig. 5e). Importantly, *LCN13* expression studies in this patient cohort revealed a highly significant correlation between *Tsc22d4* and *Lcn13* mRNA levels (Supplementary Fig. 6a), and demonstrated an overall lower expression of *LCN13* in diabetic patients as compared with non-diabetic subjects (Supplementary Fig. 6b). In addition, hepatic *LCN13* mRNA levels correlated with GIR and fasting glucose levels in humans (Supplementary Fig. 6c,d), overall recapitulating the TSC22D4-LCN13-insulin sensitization link in animal models.

## Discussion

Based on substantial side effects of existing pharmacological approaches, efficient and safe insulin sensitization and glucose control remain critical therapeutic aims to prevent diabetic late complications^20^. Our data now establish TSC22D4 as a critical node in systemic glucose metabolism and insulin sensitivity. Given its upstream regulatory function for the multi-organ enhancement of insulin sensitivity and glucose uptake, modulation of TSC22D4 function now provides novel perspectives in both the understanding of mechanisms in metabolic inter-organ communication and the development of insulin sensitizing treatment strategies.

According to the WHO, diabetes affects more that 347 million individuals these days with almost 90% suffering from insulin-resistant type 2 diabetes. For these patients, blood glucose lowering therapies are the primary treatment option. However, major classes of current anti-diabetic and/or insulin sensitizing drugs are associated with severe limitations, highlighted by the recent market withdrawal of rosiglitazone^20^.

Metformin, the most widely used first-line type 2 diabetes drug, efficiently reduces HbA1c levels. However, this reduction is often insufficient for many diabetic patients and its functional dependence on the organic cation transporter (OCT) 1 may generate a cohort of non-responders in genetic OCT1 loss-of-function carriers. Also, despite the proven efficacy of metformin, treatment is associated with gastro-intestinal side effects. Current guidelines then recommend drug combinations of metformin with sulfonylurea, thiazolidinediones, DPP-4 inhibitors or GLP-1 receptor agonists. These agents, however, are associated with severe side effects such as bone mineral density loss and increased fracture risk for thiazolidinediones and potentially dangerous hypoglycaemic episodes for sulfonylureas. Due to the progressive beta-cell dysfunction that characterizes type 2 diabetes, many diabetic patients eventually require insulin replacement therapy despite oral anti-diabetic treatment. It is tempting to speculate that the improvement of metabolic inter-tissue communication through TSC22D4 actions as demonstrated in the current manuscript may represent a particularly effective therapeutic approach in future diabetes and insulin sensitizing therapies, particularly supported by the notion of an overall conservation of the TSC22D-insulin sensitivity axis in humans.

Of note, ablation of TSC22D4 in animals did not provoke enhanced tumour formation or hepatic dysfunction within the time spans investigated thus far, indicating that TSC2D4-based long-term therapeutic application may not be associated with enhanced tumour risk as may be predicted from the potent stimulatory impact on PI3K/Akt signalling. Indeed, while potential long-term complications of TSC22D4 inhibition need to be addressed in future experimental studies in detail, our data indicate that other TSC22 family members with documented pro-tumorigenic activities^21^ were not able to compensate for the reduced levels of hepatic TSC22D4, underlining the hypothesis that TSC22D4 displays a rather unique spectrum of biological features that might spare tumorigenic potential upon long-term inhibition.

In this regard, while acute TSC22D4 inactivation in tumour-bearing animals led to hypertriglyceridemia^12^, this effect was absent upon long-term TSC22D4 inhibition in diabetic animals, indicating that elevated systemic lipid levels either represent a more transient consequence of hepatic TSC22D4 deficiency and may not compromise the beneficial effects of TSC22D4 inactivation in the long run or only occur in the wild type but not in the diabetic situation.

Overall, our data are consistent with a model in which aberrant TSC22D4 function imposes a tonic inhibition of liver insulin signalling and LCN13 secretion, thereby promoting a metabolically dysfunctional phenotype.

Interestingly, delivery of recombinant LCN13 into diabetic animals has been proven to be remarkably ineffective in terms of insulin sensitization and the restoration of glucose homeostasis^18^, particularly when compared with the marked effects of hepatic TSC22D4 knockdown as shown here, suggesting that the endogenous action of TSC22D4, comprising both LCN13 secretion as well as an additional, to-be-identified intracellular mechanism provides a particularly effective cue to control insulin sensitivity. Given the lack of efficient and safe insulin-sensitizing therapies to date, TSC22D4-mediated restoration of insulin action and thus systemic energy homeostasis under relevant disease conditions, definitely deserves further experimental exploration in the future.

## Methods

### Plasmids and RNA interference

For shRNA experiments, oligonucleotides targeting mouse *TSC22D4* (5′-GCCTGGTTGGCATTGACAACA-3′) and *LCN13* (5′-GCCGTGAGTTTAAATTCGTGA-3′), were annealed and cloned into the pENTR/U6 shRNA vector (Invitrogen). Non-specific oligonucleotides (5′-GATCTGATCGACACTGTAATG-3′) with no significant homology to any mammalian gene sequence were used as non-silencing controls in all experiments. Expression vectors for Flag-tagged TSC22D4 were generated by standard PCR-based methods and cloned into the pcDNA3.1 expression vector.). For miRNA experiments, oligonucleotides targeting mouse *TSC22D4* (5′-GACAGCGATGACGATAGTGGT-3′) and non-specific oligonucleotides (5′-AAATGTACTGCGCGTGGAGAC-3′) were cloned into pcDNA6.2-GW/EmGFP-miR (‘BLOCK-iTTM PolII miR RNAi Expression Vector Kit' (Invitrogen)). The miRNA cassette encoding a nonspecific miRNA (5′-AAATGTACTGCGCGTGGAGAC-3′) was PCR-amplified from pcDNA6.2-GW/EmGFP-miR-NC and cloned into the NotI site at the 5′ end of the EGFP coding sequence in the double stranded AAV vector pdsAAV-CMV-EGFP as described^22^.

### Recombinant viruses

Adenoviruses expressing a *TSC22D4*- or *LCN13*- or a non-specific shRNA under the control of the U6 promoter, or the TSC22D4 cDNA under the control of the CMV promoter were cloned using the BLOCK-iT Adenoviral RNAi expression system (Invitrogen). Viruses were purified by the cesium chloride method and dialysed against phosphate-buffered-saline buffer containing 10% glycerol before animal injection, as described^23^. Adeno-associated viruses encoding control or TSC22D4-specific miRNAs under the control of a hepatocyte-specific promoter were established, as described previously^22^.

### Animal experiments

Male 5–12-week old C57Bl/6 and BKS.Cg-Dock7m +/+ Lepr^db^/J (000642) mice were obtained from Charles River Laboratories and maintained on a 12 h light–dark cycle with regular unrestricted diet and free access to water. For glucose and insulin tolerance tests (GTT and ITT, respectively), mice were fasted for 4 h before intraperitoneal injections. For GTTs, fasted mice were i.p. injected with 1 mg D-glucose per g body weight. For ITTs fasted mice were i.p. injected with 1.5 U insulin per kg body weight. Unless otherwise stated, animals were fed *ad libitum* and had free access to water. For adenovirus injections, 2 × 10^9^ plaque-forming units (p.f.u.) per recombinant virus were administered via tail vein injection. For double knockdown adenovirus injections (rescue experiment) 2 × (2 × 10^9^) p.f.u. recombinant virus per treatment group were used (NC group: 4 × 10^9^ control shRNA; LCN13 group: 2 × 10^9^ LCN13 shRNA+2 × 10^9^ control shRNA; TSC22D4 group: 2 × 10^9^ TSC22D4 shRNA+2 × 10^9^ control shRNA; TSC22D4+LCN13 group: 2 × 10^9^ TSC22D4 shRNA+2 × 10^9^ LCN13 shRNA). For AAV experiments 5 × 10^11^ p.f.u. were injected via the tail vein. In high-fat diet experiments, C57Bl/6 mice were either fed a standard chow diet (10% energy from fat, Research Diets D12450B, USA) or a high-fat diet (60% energy from fat, Research Diets D12492, USA) for a period of 11 weeks. In each experiment, 4-12 animals received identical treatments and were analysed under fasted, random fed or fed conditions as indicated. Organs including liver, epididymal and inguinal fat pads, and gastrocnemius muscles were collected after specific time periods, weighed, snap-frozen and used for further analysis.

In each animal experiment, mice were randomly assigned to each group. Number of animals per group to detect biologically significant effect sizes was calculated using appropriate statistical sample size formula and indicated in the biometrical planning section of the animal licence submitted to the governing authority. Blinding was not done during animal group allocation. Animal handling and experimentation was performed in accordance with the European Union directives and the German Animal Welfare Act (Tierschutzgesetz) and approved by local authorities (Regierungspräsidium, Karlsruhe, Germany).

### Quantitative Taqman RT–PCR

Total RNA was extracted from frozen organ samples or cultured hepatocytes using QIAzol and the RNeasy (Qiagen) kit. cDNA was prepared by reverse transcription using M-MuLV enzyme and Oligo dT primer (Fermentas). cDNAs were amplified using assay-on-demand kits (Thermo Fisher Scientific) and an ABI StepOnePlus sequence detector (Applied Biosystems). RNA expression data were quantified and normalized to levels of TATA-box binding protein RNA (*Tbp*) as described^24^.

Human *TSC22D4* mRNA expression was measured by quantitative real-time RT–PCR in a fluorescent temperature cycler using the TaqMan assay, and fluorescence was detected on an ABI PRISM 7000 sequence detector (Applied Biosystems). Total RNA was isolated using TRIzol (Life Technologies), and 1 μg RNA was reverse transcribed with standard reagents (Life Technologies). From each RT–PCR, 2 μl were amplified in a 26 μl PCR reaction using the Brilliant SYBR green QPCR Core reagent kit from Stratagene) according to the manufacturer's instructions. Samples were incubated in the ABI PRISM 7000 sequence detector for an initial denaturation at 95 °C for 10 min, followed by 40 PCR cycles, each cycle consisting of 95 °C for 15 s, 60 °C for 1 min and 72 °C for 1 min. Human *TSC22D4* and *OBP2A* *(LCN13)* (determined by Hs00229526_m1 and Hs01062934_g1, respectively) (Applied Biosystems) mRNA expression was calculated relative to the mRNA expression of hypoxanthine phosphoribosyltransferase 1 (*HPRT1*), determined by a premixed assay on demand for *HPRT1* (Hs01003267_m1) (Applied Biosystems). Amplification of specific transcripts was confirmed by melting curve profiles (cooling the sample to 68°C and heating slowly to 95 °C with measurement of fluorescence) at the end of each PCR. The specificity of the PCR was further verified by subjecting the amplification products to agarose gel electrophoresis.

### Primary hepatocyte isolation and treatment

Primary mouse hepatocytes were isolated and cultured as described^25^. Briefly, male 8–12-week old C57Bl/6 mice were anesthetized by i.p. injection of 100 mg kg^−1^ body weight ketamine hydrochloride and 5 mg kg^−1^ body weight xylazine hydrochloride. After opening the abdominal cavity, the liver was perfused at 37 °C with HANKS I buffer via the portal vein for 5 min and subsequently with HANKS II buffer for 5–7 min until disintegration of the liver structure was observed. The liver capsule was removed and the cell suspension was filtered through a 100 μm mesh. The cells were washed and, subsequently, viability of cells was determined by trypan blue staining. 1 × 10^6^ living cells per well were seeded on collagen I-coated 6-well plates. After 24 h, cells were infected with recombinant adenoviruses at a multiplicity of infection of 100. Primary mouse hepatocytes were either treated with PBS or insulin at a concentration of 100 nM per well for 10 min.

### Gene expression profiling

Gene expression profiling was performed for liver extracts from control or TSC22D4 shRNA adenovirus-treated C57Bl/6 mice; control, *LCN13*, TSC22D4, TSC22D4 plus LCN13 adenovirus-treated BKS.Cg-Dock7m +/+ Lepr^db^/J (000642) mice. Gene expression profiling was performed using arrays of Mouse Genome 430 2.0 and murine MoGene-2_0-st-type from Affymetrix. Biotinylated sense-strand DNA was then prepared according to the Affymetrix standard labeling protocol. Afterwards, the hybridization on the chip was performed on a GeneChip Hybridization oven 640, then dyed in the GeneChip Fluidics Station 450 and thereafter scanned with a GeneChip Scanner 3000. All of the equipment used was from the Affymetrix-Company. A Custom CDF (Microarray Lab, Dept. of Psychiatry/Molecular and Behavioral Neuroscience Institute, University of Michigan, MI, USA) Version 17 with Entrez-IDs based gene definitions was used to annotate the arrays. Raw data were log2 transformed, quantile-normalized and RMA background corrected. Differential gene expression was analysed based ANOVA, using a commercial software package SAS JMP7 Genomics, version 6, from SAS (SAS Institute, Cary, NC, USA). A false positive rate of *a*=0.05 with FDR correction was taken as the level of significance. Gene set enrichment analysis (GSEA) was used to determine whether defined lists (or sets) of genes exhibit a statistically significant bias in their distribution within a ranked gene list. Pathways belonging to various cell functions such as cell cycle or apoptosis were obtained from public external databases (KEGG, http://www.genome.jp/kegg/).

### Chromatin immunoprecipitation

Mouse liver was crosslinked in 1% formaldehyde for 15 min, followed by quenching with 1/20 volume of 2.5 M glycine solution, and two washes with 1 × PBS. Nuclear extracts were prepared by dounce homogenization in cell lysis buffer (50 mM Tris, 150 mM NaCl, 1 mM EDTA, 1% SDS, protease inhibitor cocktail). Chromatin fragmentation was performed by sonication in ChIP-SDS lysis buffer (50 mM HEPES, 1% SDS, 10 mM EDTA, pH7.5) using the Bioruptor (Diagenode), and Flag-tagged or endogenous TSC22D4proteins were immunoprecipitated in ChIP dilution buffer (50 mM Tris, 155 mM NaCl, 1.1% Triton X-100, 0.11% Na-deoxycholate, 1 mM PMSF and complete protease 3 inhibitor tablet, pH7.5) using an anti-Flag (#F3165, Sigma) or two distinct TSC22D4antibodies (ab76915, Abcam,; SAB2105343, Sigma-Aldrich), respectively. Cross-linking was reversed overnight at 65 °C, and DNA isolated using phenol/chloroform/isoamyl alcohol. Precipitated DNA was analysed using Illumina Hi-Seq 2000 for sequencing or real-time PCR against specific regions within the *LCN13* locus. Each sample was measured in duplicates and the mean Ct-value was calculated. Each mean value was then normalized to the Ct-value of the respective input DNA Ct-value for the same PCR assay (ΔCt) according to the following formula: ΔCt=(Ct[ChIP]–(Ct[Input]–Log2(input dilution factor))) with input dilution factor=1/(fraction of input saved). Fraction of input equals the amount of input protein that was used for DNA purification divided by the amount that was used to make the IP. Next the % input was calculated for each ChIP fraction (linear conversion of the normalized ChIP ΔCt) by using the following formula: % input=2(−ΔCt) which was eventually normalized to background (mock IP) Ct value using the following formula: ΔΔCt[ChIP/Mock]=ΔCt[ChIP] –ΔCt[Mock]. The IP fold enrichment above the sample specific background was finally calculated as follows: IP fold enrichment=2(−ΔΔCt[ChIP/Mock]). This calculation allows the ChIP data to be normalized to both mock input as well as background levels. ChIP primers were as follows:

Peak1: fragment size: 225; forward 5′-GGAGAGCACAGATGGCATCTACCTA-3′; reverse 5′-TCAAGCCTTTGAGAGTTCCATGCCA-3′

Peak2: fragment size: 245 bp; forward 5′-GACGTGTAGACCTCAATGAGTGCAT-3′; reverse 5′-CCAGATCCCAGAGTACTGTAGGAGA-3′

Peak3: fragment size 216 bp; forward 5′-CTTCTGAAGCATGCAGGGCTGGATCAT-3′; reverse 5′-TTGGGTGAGGCAGGCACACAGTAT-3′

Peak4: fragment size 207 bp; forward 5′-CACTTGCTGCTCTTCCAGAGGACTCAA-3′; reverse 5′-TGGCCCTCATAAGCAGAGTCTAGATT-3′

### Cell culture and transient transfections

Plasmids carrying TSC22D4-binding sites within the *LCN13* locus were cloned into pGL3prom Luc vector (Promega) by standard PCR using primer pairs used for the ChIP-PCR. Murine Hepa 1-6 cells (ATCC) were maintained in DMEM-high glucose (#11995, Gibco) supplemented with 10% heat-inactivated fetal bovine serum (FBS) at 37°C in a humidified atmosphere containing 5% CO_2_. For luciferase assay transfections, calcium phosphate precipitation method was used as described (200 ng reporter plasmid per well)^26^. Where indicated, expression plasmids encoding TSC22D4-specific or non-specific shRNA were co-transfected (400 ng plasmid per well, respectively). Cells were harvested 48 h after transfection for luciferase assays. The luciferase activity was normalized to beta galactosidase activity.

C2C12 myoblasts (ATCC) were differentiated into mature myotubes as described^27^. Mature myotubes were incubated with 200 nM recombinant LCN13 from two different sources (BioCat and R&D Systems,) for 3 h and cells were stimulated with insulin (30 nM) for 15 min before harvesting for western blot analysis as described below.

### Cell lysis and protein analysis

Proteins were extracted from frozen organ samples or cultured cells following lysis in ice cold lysis buffer A (50 mM Tris pH 7.2, 150 mM NaCl, 1% NP-40, 0.5 % Triton X, 1 mM EDTA, 1 mM Na_3_V0_4_, 1 mM NaF, 1 μg ml^−1^ pepstatin A and 1 mM DTT). Western blot assays were performed using antibodies specific for TSC22D4 (home-made TSC22D4 polyclonal antibody was generated and purified by PINEDA Antibody Service (Berlin, Germany), LCN13 (AF7974, R&D Systems; sc-164876, Santa Cruz Antibodies), p-Akt (S473) (#9271, CST), p-Akt (T308) (#13038, CST), Akt (#9272 ,CST), p-GSK3 beta (S9) (#89336,CST), GSK3 beta (#9315, CST), Glut4 (#07-1404, Millipore), VCP (ab11433, Abcam), p-Foxo1 (S256) (#9461, CST), Foxo1 (#2880 CST). For TSC22D4 antibody generation, a TSC22D4 peptide was coupled via an N-terminal cysteine to the carrier protein maleimide-activated mariculture keyhole limpet hemocyanin (KLH). Antipeptide antibodies were affinity purified from rabbit serum by positive selection on antigen peptide. Immunoblots were developed using ECL reagent (#RPN2106, Amersham) or ECL Prime for weaker signals (#RPN2236, Amersham) and detected digitally with a BioRad ChemiDoc Imaging System or with autoradiography films. For digitally acquired images quantification was performed using the ImageLab software. Scanned autoradiography films were quantified using the ImageJ software. For some figures, unrelated lanes were cropped out and flanking lanes were put side by side using Adobe Photoshop. We indicated this kind of a modification with a thin, vertical black line. Full size images are provided in Supplementary Fig. 7. Cell lines were regularly tested for mycoplasma contamination as required by in-house policies.

### Blood metabolites

Serum levels of glucose and triglycerides (TAGs) were determined using an automatic glucose monitor (One Touch, Lifescan, Germany) or commercial kits (Sigma-Aldrich, Germany; RANDOX, Northern Ireland; WAKO, Germany), respectively. Insulin levels were determined using a mouse insulin enzyme-linked immunosorbent assay (ELISA) (Mercodia, Sweden). HOMA-IR was calculated using the following formula: HOMA-IR (mmol l^−1^ × μU ml^−1^)=fasting glucose (mmol l^−1^) × fasting insulin (μU ml^−1^)/22.5. HOMA-ISI was calculated using the following formula=10,000/(SQRT (fasting glucose (mmol l^−1^) × fasting insulin (μU ml^−1^) × (mean glucose (mmol l^−1^) × mean insulin (μU ml^−1^)).

For HbA1c determination 20 μl of whole blood were homogenized in 80 μl ice-cold A. dest., incubated on ice for 10 min and centrifuged at 15,000*g* for 10 min at 4 °C. Ten microlitre of the resulting supernatant were transferred to HPLC vials containing 125 μl of 20 mM Bis-Tris, 2 mM KCN (pH 6.9). The HbA1c fraction (HPLC: PolyCAT A column (35 mm × 4.6 mm, 3 μ, 1,500 Å) from PolyLC Inc. (USA) on a LaChrom Elite HPLC System from Hitachi High-Technologies Corporation (Japan) was identified by comparison to the retention time of pure standard of haemoglobin. The HbA1c fraction was quantified as % of non-glycated haemoglobin by calculating the ratio of HbA1c and Hb0 peak areas.

### Tissue lipid extraction

Hepatic lipids were extracted as described previously^28^, and TAG and total cholesterol content was determined using commercial kits as above. Values were calculated as milligrams (TAG and cholesterol) per gram wet tissue.

### Human studies

We investigated *TSC22D4* mRNA expression in liver tissue samples obtained from 66 extensively characterized Caucasian obese and lean men and women who underwent open abdominal surgery for Roux en Y bypass, sleeve gastrectomy, elective cholecystectomy or explorative laparotomy. With oral glucose tolerance tests, we identified individuals with type 2 diabetes (*n*=26) or normal glucose tolerance (*n*=40). The phenotypic characterization of the cohort has been extensively described previously^29^. Insulin sensitivity was assessed using the euglycaemic-hyperinsulinemic clamp method as described previously^30^. All baseline blood samples were collected between 8:00 and 10:00 after an overnight fast. All study protocols have been approved by the Ethics Committee of the University of Leipzig (363-10-13122010 and 017-12-230112). All participants gave written informed consent before taking part in the study.

### Statistical analysis

For each experiment, means and s.e.m. or s.d. (specified in Figure Legends) of parameters measured were determined. Statistical analyses were performed using Student's *t*-test in one-factorial designs. Correlation was determined using Pearson's correlation coefficient; F-test was applied to determine significance. For multi-factorial study designs, two-way ANOVA was used when appropriate. Holm–Sidak post hoc was applied when significant differences were found with an overall significance level=0.05. All analyses were carried out with SigmaPlot v.12 software or GraphPad Prism.

### Data availability

The authors declare that the data supporting the findings of this study are available within the article and its supplementary information files, or available from the corresponding author upon reasonable request.

## Additional information

**How to cite this article:** Ekim Üstünel, B. *et al*. Control of diabetic hyperglycaemia and insulin resistance through TSC22D4. *Nat. Commun.* **7,** 13267 doi: 10.1038/ncomms13267 (2016).

**Publisher's note:** Springer Nature remains neutral with regard to jurisdictional claims in published maps and institutional affiliations.

## Supplementary Material

Supplementary Information Supplementary Figures 1-7, Supplementary Table 1
