# Supplementary material for: Control of diabetic hyperglycaemia and insulin resistance through TSC22D4
Source: Nat Commun. 2016 Nov 9;7:13267. doi: 10.1038/ncomms13267 (PMC5105165; doi:10.1038/ncomms13267)
Supplement: Supplementary Information — Supplementary Figures 1-7, Supplementary Table 1 [file ncomms13267-s1.pdf]

Supplementary Figure 1

a

Term	Count	p-value	Benjamini
Cell cycle	44	2.25E-8	4.30E-6
p53 signaling pathway	25	1.54E-5	3.4E-5
Oocyte meiosis	35	1.67E-5	1.9E-4
Insulin signaling pathway	39	3.25E-5	1.6E-4
Ubiquitin mediated proteolysis	36	2.85E-4	2.0E-4
Lysosome	32	4.99E-4	4.30E-6
Apoptosis	24	0.00207	3.4E-5
Pathways in cancer	66	0.00214	1.9E-4
Biosynthesis of unsaturated fatty acids	11	0.00290	1.6E-4
Progesterone-mediated oocyte maturation	23	0.00342	2.0E-4
Vitamin B6 metabolism	5	0.00495	2.0E-4

b

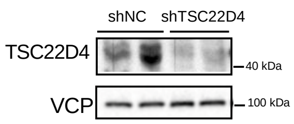

c d

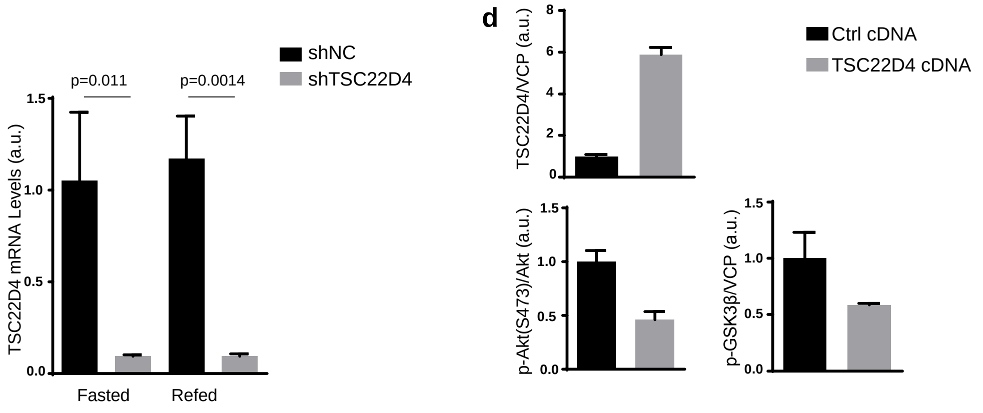

Supplementary Figure 1: (**a**) Most significantly regulated pathways in mouse primary hepatocytes treated with either control or *Tsc22d4* shRNA adenovirus based on KEGG-pathway analysis. (**b**) Western blot analysis of liver extracts from random fed representative control (shNC) or *Tsc22d4* (sh *Tsc22d4*) shRNA adenovirus–injected C57Bl/6 mice 7 days after injection using Vcp and Tsc22d4 antibodies. **(c)** Quantitative PCR analysis of *Tsc22d4* mRNA levels in the livers of mice as in Fig. 1c. Statistical analysis: StudentStudent`s t-test. (**d**) Quantification of the immunoblots shown in Fig. 1d normalized to total protein levels or loading control Vcp. Error bars indicate standard deviation (s.d.).

Supplementary Figure 2

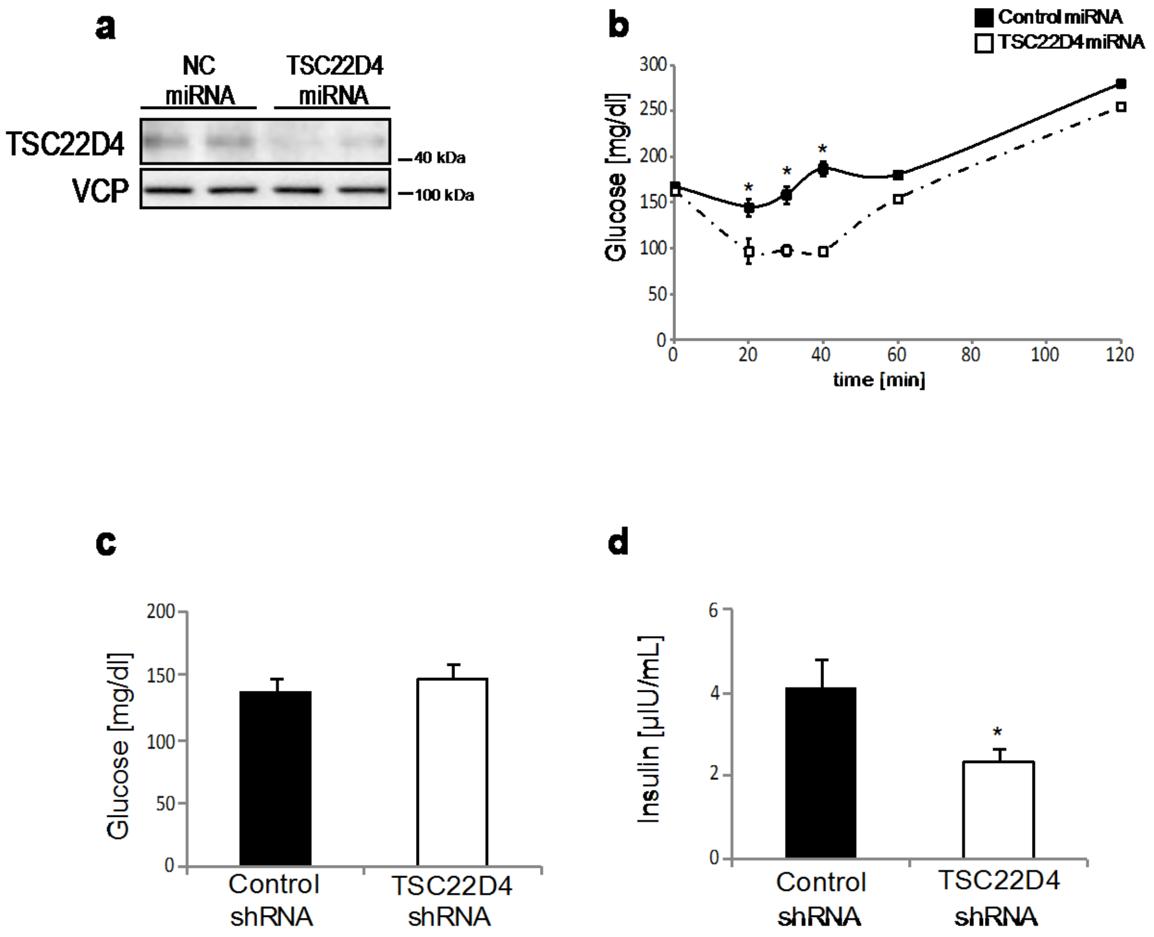

Supplementary Figure 2: (**a**) Western Blot analysis of liver extracts from random fed control (NC miRNA) or *Tsc22d4* (*Tsc22d4* miRNA) miRNA AAV–injected C57Bl/6 mice using Vcp and Tsc22d4 antibodies (**b**) Insulin tolerance test of same mice as in (a) (means ± s.e.m, $n = 5$). (**c**) Glucose levels of 6 h refed control or *Tsc22d4* shRNA adenovirus–injected C57Bl/6 mice 7 days after injection (means ± s.e.m, $n \geq 6$). (**d**) Serum insulin levels in same mice as in (c). Statistical analysis for b-d: StudentStudent`s t-test, *: $p \leq 0.05$.

Supplementary Figure 3

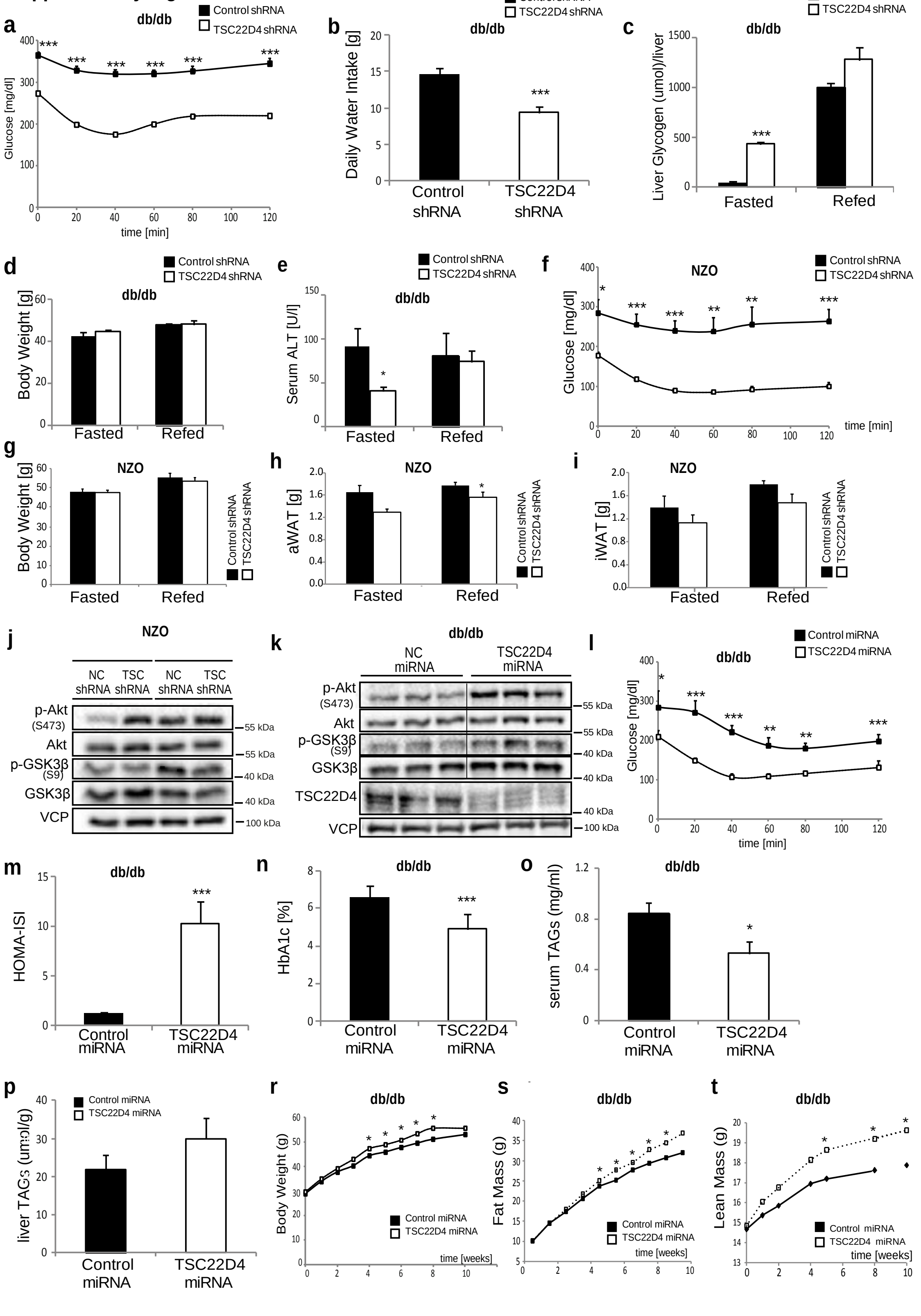

Supplementary Figure 3: (**a**) Insulin tolerance test of control or *Tsc22d4* shRNA adenovirus–injected db/db mice 1 week after injection. Insulin was i.p. injected at a concentration of 1.5U Insulin kg^{-1} body weight (means ± s.e.m, $n \geq 6$). (**b**) Daily water intake of same mice as in (a). (**c**) Liver glycogen levels of same mice as in (a). (**d**) Body weight of same mice as in (a). (**e**) Serum alanine-aminotransferase (ALT = GPT) levels of same mice as in (a). (**f**) Insulin tolerance test of control or *Tsc22d4* shRNA adenovirus–injected NZO mice 1 week after injection (means ± s.e.m, $n \geq 6$). (**g**) Body weight (**h**) abdominal fat mass (**i**) inguinal fat mass of same mice as in (f) (**j**) Representative western blot analysis of liver extracts from control (NC shRNA) or *Tsc22d4* (TSC shRNA) shRNA adenovirus–injected NZO mice; same mice as in (f) using indicated antibodies. (**k**) Western Blot analysis of liver extracts of control (NC miRNA) or *Tsc22d4* (*Tsc* miRNA) miRNA AAV–injected (at 5 weeks of age) db/db mice 7 weeks after injection using indicated antibodies (means ± s.e.m, $n \geq 6$). (**l**) Insulin tolerance test in same mice as in (k). (**m**) HOMA insulin-sensitivity index (HOMA-ISI) of same mice as in (k). (**n**) HbA1c levels of same mice as in (k). (**o**) Serum triglycerides (TAGs) of the same mice as in (k). (**p**) Liver TAGs of the same mice as in (k). (**r**) Body weight (**s**) fat mass (**t**) lean mass development of the same mice as in (k). Statistical analysis: Student`s t-test, *: $p \leq 0.05$; **: $p \leq 0.01$; ***: $p \leq 0.001$.

a

Supplementary Figure 4

GeneName	changefoldexpression	-log10(p-value) for Diff
LCN13	11.48	5.09
Elovl6	7.02	3.86
Hes6	6.53	2.72
Mfsd2	6.39	2.18
Colec10	6.38	2.60
Fasn	5.38	3.50
Stard4	5.00	3.42
Acly	4.97	3.64
Polr3g	4.88	3.84
Lpin	4.63	6.33

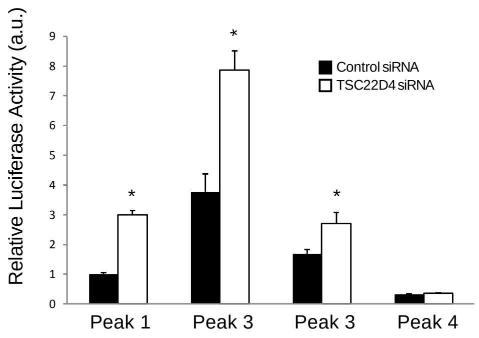

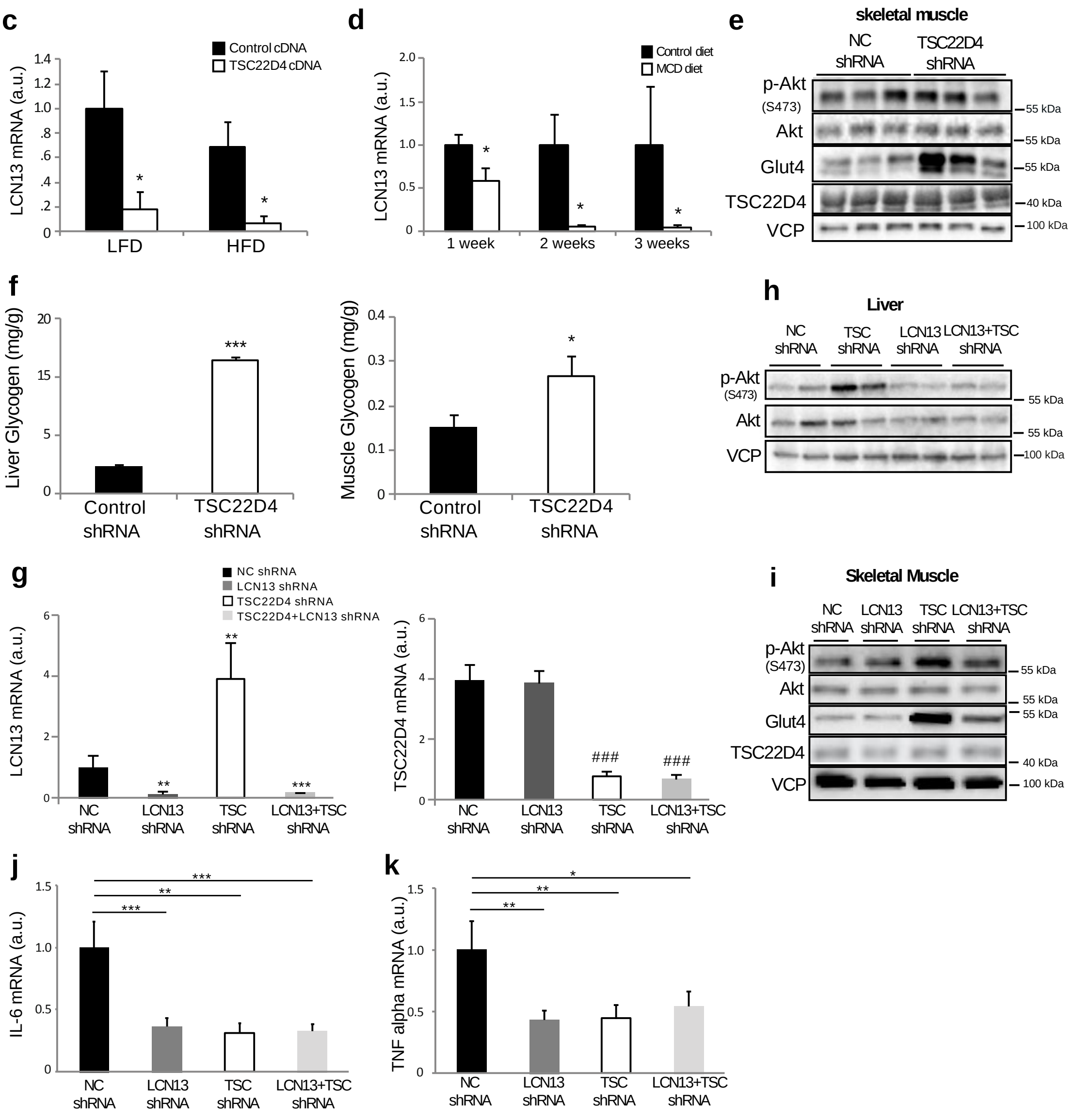

Supplementary Figure 4: (**a**) Most significantly upregulated genes in the liver based on microarray data analysis of random fed control or *Tsc22d4* shRNA adenovirus–injected C57Bl/6 mice 7 days after injection. (**b**) Luciferase activity assay in transiently transfected of Hepa 1-6 hepatoma cells with luciferase reporter plasmids carrying Tsc22d4 binding sites belonging to *Lcn 13* locus (200 ng/well). Co-transfection of control and *Tsc22d4*-specific shRNA plasmids (400 ng/well) indicated (means ± SEM, n = 3-6). (**c**) Quantitative PCR analysis of *Lcn 13* in livers of empty control (control cDNA) or Flag- *Tsc22d4* (*Tsc22d4*cDNA) cDNA adenovirus-injected random fed C57Bl/6 mice that were fed either a LFD or HFD for 11 weeks (means ± s.e.m, n = 5). (**d**) Quantitative PCR analysis of *Lcn 13* in livers of C57Bl/6 mice fed a standard chow diet or a methionine and choline deficient diet for a period of 1, 2 and 4 weeks (means ± s.e.m, n = 5). (**e**) Representative western blot analysis of skeletal muscle extracts from fasted (Glut4, TSC22D4, and VCP) or refed (p-Akt and Akt) control (NC shRNA) or *Tsc22d4* (*Tsc22d4*shRNA) shRNA adenovirus–injected db/db mice using indicated antibodies (**f**) Liver (left) and muscle (right) glycogen levels of control or *Tsc22d4* shRNA adenovirus–injected C57Bl/6 mice 7 days after injection (means ± s.e.m, $n \geq 6$). (**g**) Quantitative PCR analysis of *Lcn 13* (left) and *Tsc22d4* (right) in livers of control (NC shRNA), *Lcn 13* shRNA, *Tsc22d4* shRNA, or *Tsc22d4* plus *Lcn 13* shRNA adenovirus–injected db/db mice 1 week after injection (means ± s.e.m, $n \geq 6$; * indicates significant differences of *Lcn 13* mRNA expression compared to control group; # indicates significant differences of *Tsc22d4* mRNA expression compared to control group). (**h**) Western blot analysis of liver extracts from control (NC shRNA), *Lcn 13* shRNA, *Tsc22d4* shRNA or *Tsc22d4* plus *Lcn 13* shRNA adenovirus–injected db/db mice; same mice as in (e) using indicated antibodies. (**i**) Western blot analysis of skeletal muscle extracts from same mice as in (h) using indicated antibodies. (**j) and (k**)

Quantitative PCR analysis of *Interleukin-6* (*Il-6*) (j) and *Tnf-alpha* (k) in livers of control (NC shRNA), *Lcn 13* shRNA, *Tsc22d4* shRNA, or *Tsc22d4* plus *Lcn 13* shRNA adenovirus–injected db/db mice 1 week after injection (means ± s.e.m, $n \geq 6$; * indicates significant differences of *Il-6* or *Tnf alpha* mRNA expression compared to control group). Statistical analysis: Student`s t-test, *: $p \leq 0.05$ **: $p \leq 0.01$; ***: $p \leq 0.001$. b-d and g-k: y-axis is in arbitrary units.

Supplementary Figure 5

a

NAME	FDR q-val
MMU05144 MALARIA	0,000
MMU04512 ECM-RECEPTOR INTERACTION	0,000
MMU04670 LEUKOCYTE TRANSENDOTHELIAL MIGRATION	0,001
MMU05323 RHEUMATOID ARTHRITIS	0,001
MMU04514 CELL ADHESION MOLECULES (CAMS)	0,001
MMU04510 FOCAL ADHESION	0,002
MMU05100 BACTERIAL INVASION OF EPITHELIAL CELLS	0,002
MMU04142 LYSOSOME	0,002
MMU00640 PROPANOATE METABOLISM	0,002
MMU04540 GAP JUNCTION	0,002
MMU03320 PPAR SIGNALING PATHWAY	0,003
MMU04145 PHAGOSOME	0,003
MMU05322 SYSTEMIC LUPUS ERYTHEMATOSUS	0,004
MMU00280 VALINE, LEUCINE AND ISOLEUCINE DEGRADATION	0,008
MMU00100 STEROID BIOSYNTHESIS	0,013
MMU04640 HEMATOPOIETIC CELL LINEAGE	0,014
MMU05020 PRION DISEASES	0,014
MMU00970 AMINOACYL-TRNA BIOSYNTHESIS	0,015
MMU05140 LEISHMANIASIS	0,016
MMU05143 AFRICAN TRYPANOSOMIASIS	0,016
MMU00040 PENTOSE AND GLUCURONATE INTERCONVERSIONS	0,017
MMU04062 CHEMOKINE SIGNALING PATHWAY	0,020
MMU04974 PROTEIN DIGESTION AND ABSORPTION	0,020
MMU00350 TYROSINE METABOLISM	0,021
MMU00340 HISTIDINE METABOLISM	0,032
MMU05145 TOXOPLASMOSIS	0,033
MMU00531 GLYCOSAMINOGLYCAN DEGRADATION	0,034
MMU04380 OSTEOCLAST DIFFERENTIATION	0,035
MMU05142 CHAGAS DISEASE (AMERICAN TRYPANOSOMIASIS)	0,037
MMU04060 CYTOKINE-CYTOKINE RECEPTOR INTERACTION	0,038
MMU04115 P53 SIGNALING PATHWAY	0,038
MMU00510 N-GLYCAN BIOSYNTHESIS	0,039
MMU04360 AXON GUIDANCE	0,042
MMU00561 GLYCEROLIPID METABOLISM	0,044
MMU04612 ANTIGEN PROCESSING AND PRESENTATION	0,044
MMU05146 AMOEBIASIS	0,046
MMU04964 PROXIMAL TUBULE BICARBONATE RECLAMATION	0,048
MMU00030 PENTOSE PHOSPHATE PATHWAY	0,049

b

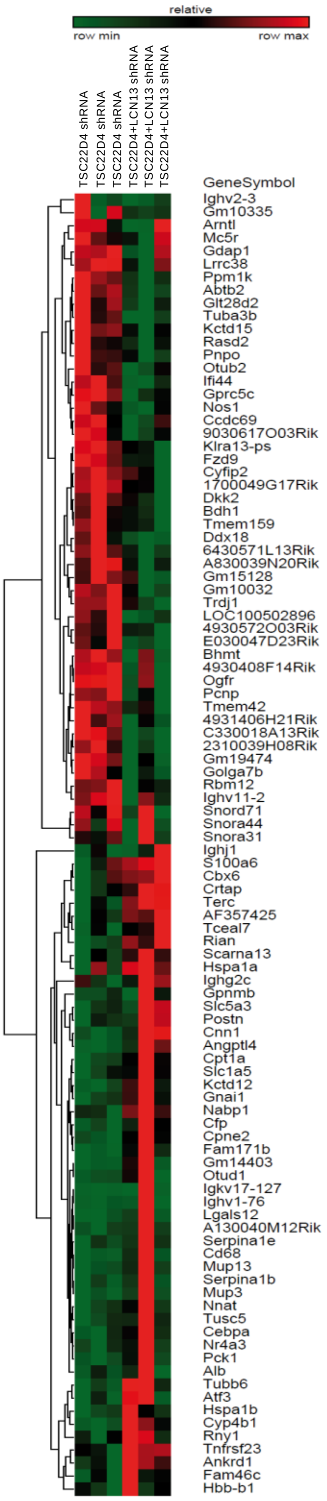

c

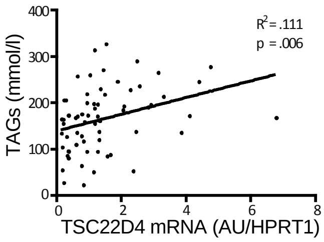

d

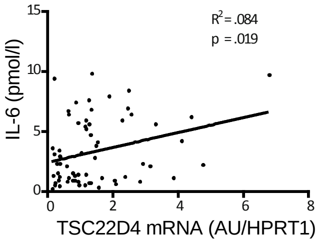

e

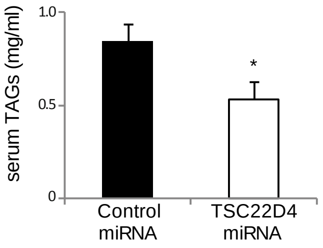

Supplementary Figure 5: (**a**) Most significantly regulated pathways of skeletal muscle extracts of *Tsc22d4* shRNA or *Tsc22d4* plus *Lcn 13* shRNA adenovirus–injected db/db mice 1 week after injection (means ± s.e.m, *n* = 3) based on KEGG-pathway analysis. (**b**) Heat map of differentially regulated genes of skeletal muscle extracts from the same animals as in (a). (**c**) Correlation of human liver expression of *TSC22D4* mRNA and serum TAGs of patients with type 2 diabetes (*n* = 26) or normal glucose tolerance (*n* = 40). (**d**) Correlation of human liver expression of *TSC22D4* mRNA and serum IL-6 levels in same patients as in (c). (**e**) Serum TAGs in control or *Tsc22d4* miRNA AAV–injected db/db mice 10 weeks after injection. Statistical analysis for c-d: Pearson correlation coefficient, e: Student`s t-test, *: $p \leq 0.05$.

Supplementary Figure 6

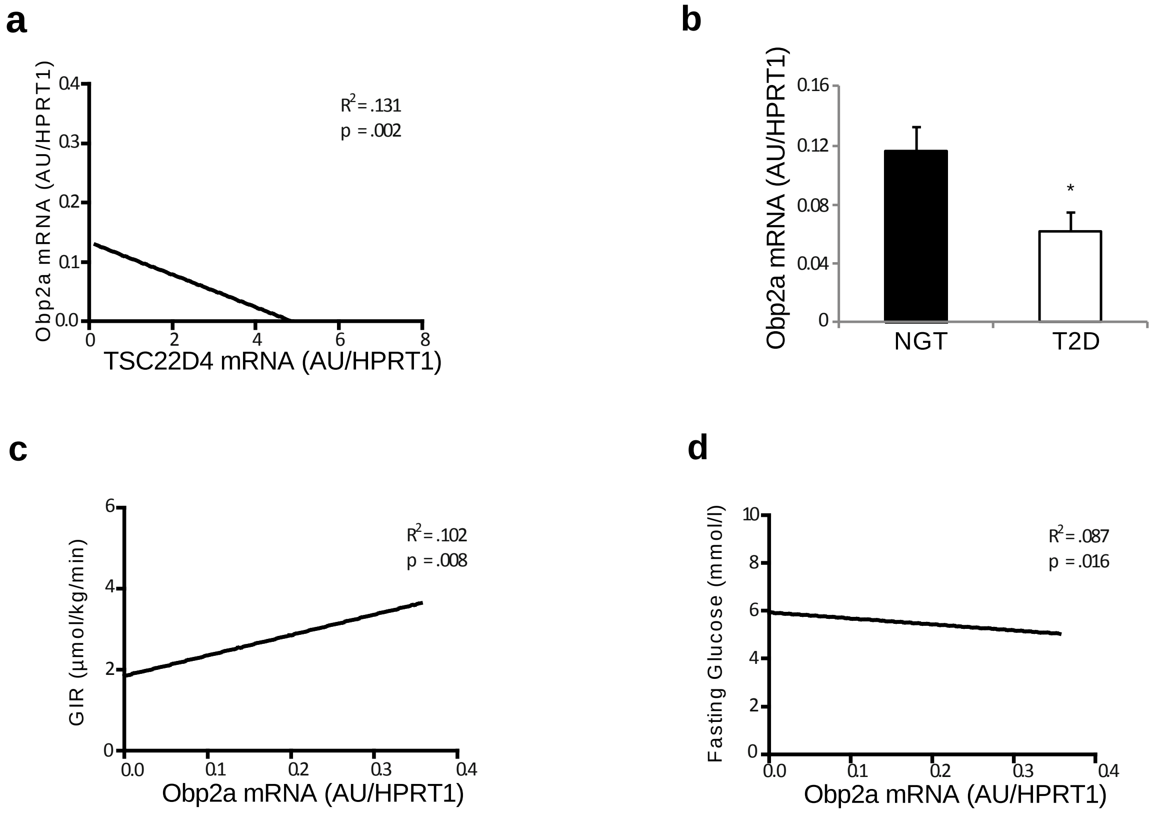

Supplementary Figure 6: (**a**) Correlation of human liver expression of *LCN13* (*OBP2A*) and *TSC22D4* mRNA levels in patients with type 2 diabetes (n = 26) or normal glucose tolerance (n = 40). (**b**) Quantitative PCR analysis of *LCN13* (*OBP2A*) mRNA expression in livers of patients with type 2 diabetes (T2D, n = 26) or normal glucose tolerance (NGT, n = 40). (**c**) Correlation of human liver expression of *LCN13* (*OBP2A*) mRNA and glucose infusion rate (GIR) during hyperinsulinemic-euglycemic clamp study in the same patients as in (a). (**d**) Correlation of hepatic expression of *LCN13* (*OBP2A*) mRNA and fasting plasma glucose levels in the same patients as in (a). Statistical analysis for a, c, d: Pearson correlation coefficient, b: Student`s t-test, *: $p \leq 0.05$

Supplementary Figure 7

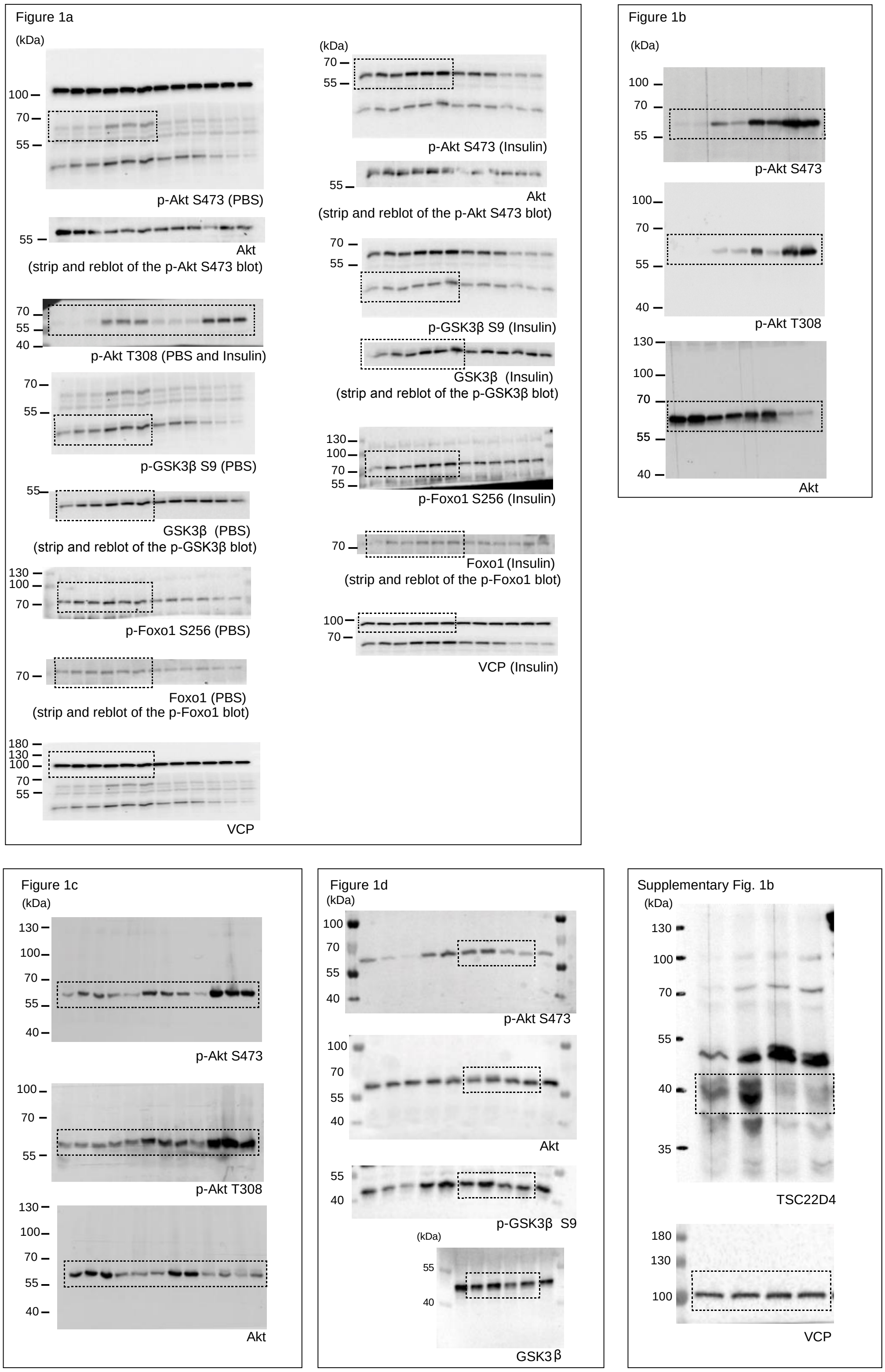

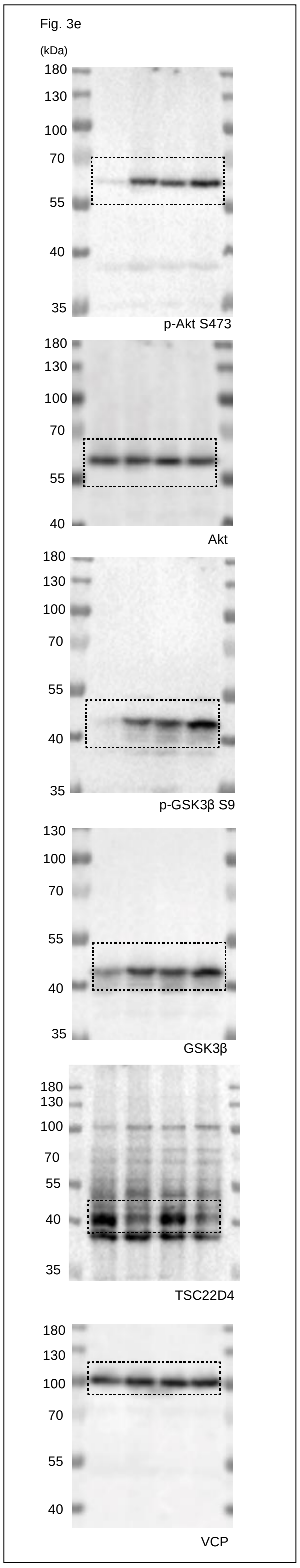
Fig. 3e
(kDa)
180
130
100
70
55
40
35
p-Akt S473
Akt
p-GSK3β S9
GSK3β
TSC22D4
VCP

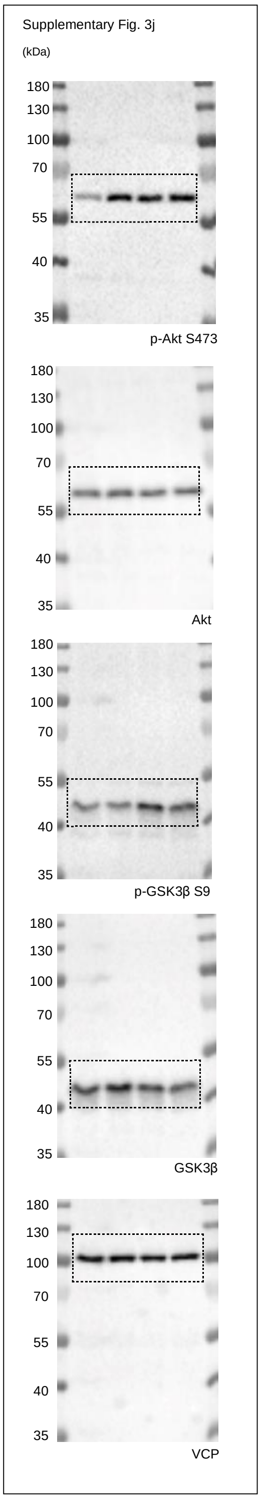
Supplementary Fig. 3j
(kDa)
180
130
100
70
55
40
35
p-Akt S473
Akt
p-GSK3β S9
GSK3β
VCP

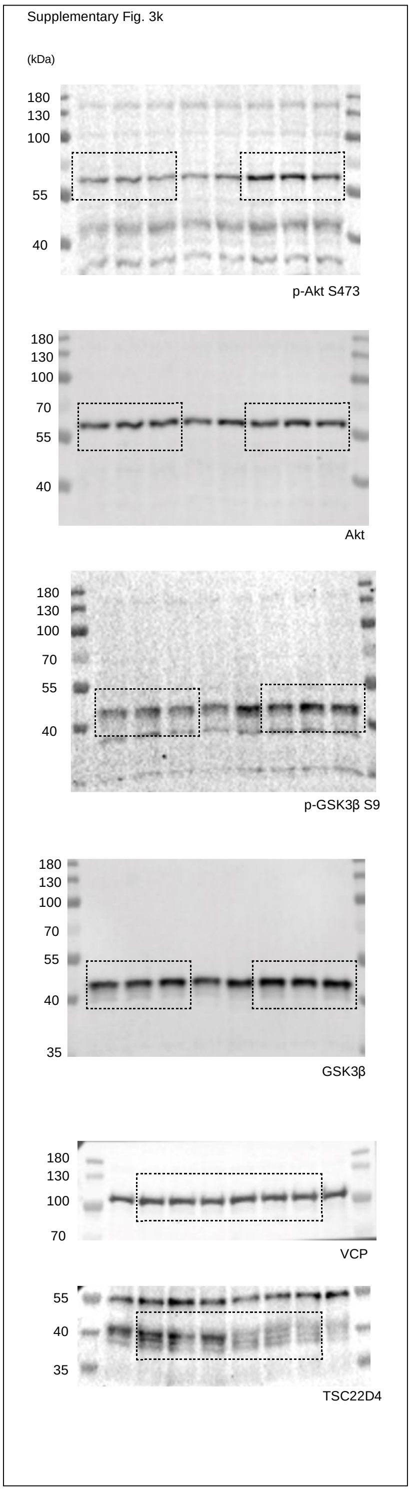
Supplementary Fig. 3k
(kDa)
180
130
100
70
55
40
35
p-Akt S473
Akt
p-GSK3β S9
GSK3β
VCP
TSC22D4

Part 2 of 3

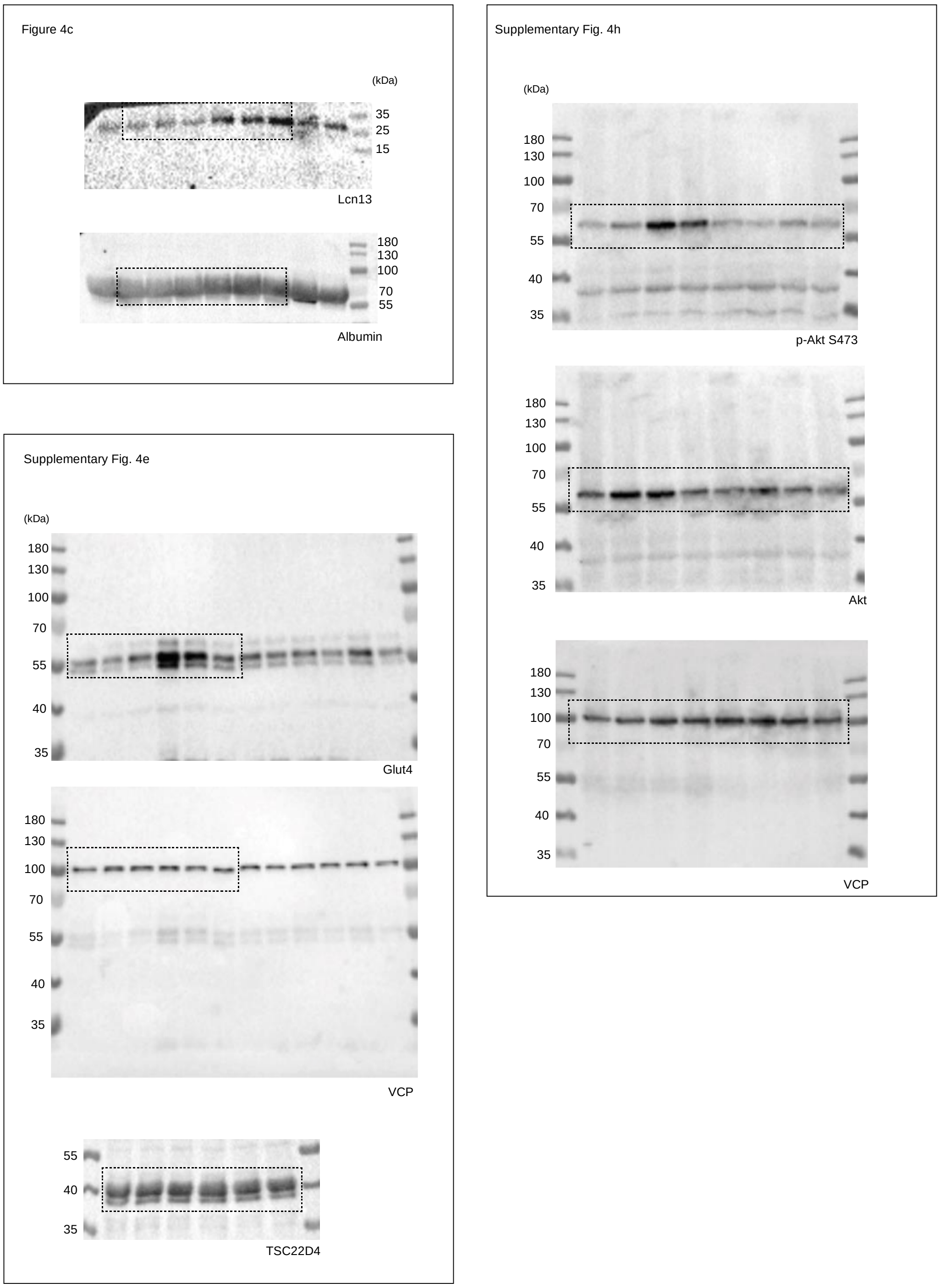

Part 3 of 3

Supplementary Figure 7: Full size images from main and supplementary figures.

Supplemental Table 1

	**Type 2 Diabetes**	**Normal glucose tolerance**	**P value**
N =	26	40	
Gender (female/male)	12/14	17/23	
BMI (kg/m²)	33.5 ± 6.3	31.3 ± 6.6	0.09
Glucose infusion rate (µmol/kg/min), Clamp	36.9 ± 21.6	71.7 ± 28.6	<0.01
Fasting plasma glucose (mmol/l)	6.38 ± 0.79	5.25 ± 0.45	<0.01
Triglycerides (mmol/l)	2.38 ± 0.6	1.53 ± 0.7	<0.01
Interleukin-6 (pg/ml)	5.33 ± 2.5	1.96 ± 1.9	<0.05

Supplementary Table 1. Characteristics of the human study cohort consisting of patients with type 2 diabetes (n = 26) or normal glucose tolerance (n = 40) regarding gender, body-mass-index (BMI), glucose-infusion rate determined by hyperinsulinemic-euglycemic clamp study, fasting plasma glucose, serum TAGs and serum levels of IL-6. Statistical analysis: StudentStudent`s t-test.
